# Honokiol ameliorates angiotensin II‐induced cardiac hypertrophy by promoting dissociation of the Nur77–LKB1 complex and activating the AMPK pathway

**DOI:** 10.1111/jcmm.18028

**Published:** 2023-11-20

**Authors:** Xiaoyan Lin, Hailin Zhang, Yong Chu, Yuze Zhang, Changsheng Xu, Hong Xie, Qinyun Ruan, Jinxiu Lin, Chun‐Kai Huang, Dajun Chai

**Affiliations:** ^1^ Echocardiological Department, The First Affiliated Hospital Fujian Medical University Fuzhou China; ^2^ Cardiovascular Department, Fujian Institute of Hypertension, The First Affiliated Hospital Fujian Medical University Fuzhou China; ^3^ Cardiovascular Department, National Regional Medical Center, Binhai Branch of the First Affiliated Hospital Fujian Medical University Fuzhou China

**Keywords:** angiotensin II, cardiac hypertrophy, honokiol, LKB1, Nur77

## Abstract

Pathological cardiac hypertrophy is a key contributor to heart failure, and the molecular mechanisms underlying honokiol (HNK)‐mediated cardioprotection against this condition remain worth further exploring. This study aims to investigate the effect of HNK on angiotensin II (Ang II)‐induced myocardial hypertrophy and elucidate the underlying mechanisms. Sprague–Dawley rats were exposed to Ang II infusion, followed by HNK or vehicle treatment for 4 weeks. Our results showed that HNK treatment protected against Ang II‐induced myocardial hypertrophy, fibrosis and dysfunction *in vivo* and inhibited Ang II‐induced hypertrophy in neonatal rat ventricular myocytes *in vitro*. Mechanistically, HNK suppressed the Ang II‐induced Nur77 expression at the transcriptional level and promoted ubiquitination‐mediated degradation of Nur77, leading to dissociation of the Nur77–LKB1 complex. This facilitated the translocation of LKB1 into the cytoplasm and activated the LKB1‐AMPK pathway. Our findings suggest that HNK attenuates pathological remodelling and cardiac dysfunction induced by Ang II by promoting dissociation of the Nur77–LKB1 complex and subsequent activation of AMPK signalling. This study uncovers a novel role of HNK on the LKB1‐AMPK pathway to protect against cardiac hypertrophy.

## 1 INTRODUCTION

Pathological hypertrophy of the myocardium, which is a significant predisposing factor for heart failure, is a common response to various intrinsic (e.g. angiotensin II, Ang II) or extrinsic stimuli.^1^ This hypertrophy is primarily characterized by the enlargement of fully differentiated myocytes, which occurs due to the increase in protein synthesis levels, size and sarcomere organization. This process is associated with the reactivation of the fetal gene program and is triggered by the collective action of various signalling proteins that transactivate pro‐hypertrophic genes.

The mammalian target of rapamycin (mTOR) is a serine/threonine‐protein kinase that plays a crucial role in coordinating nutrient availability and growth factor signalling during cell metabolism and growth. mTORC1 and mTORC2 are two different complexes of mTOR that regulate protein synthesis and degradation.^2^ mTORC1 activity increases during the progression of both physiological and pathological hypertrophy, which is stimulated by various metabolic, mechanical and biochemical signals. This increase in activity leads to a boost in ribosomal protein production (mRNA translation) by activating ribosomal protein S6 kinase β1 (S6K1) and suppressing eIF4E‐binding protein 1 (4EBP1), thus enabling unrestricted cap‐dependent translation via eIF4E.^3^ AMP‐activated protein kinase (AMPK) is a crucial energy balance sensor in mammalian cells,^4^, ^5^ and liver kinase B1 (LKB1) acts as its key upstream kinase, mediating the phosphorylation of AMPK at Thr172.^6^ Under normal physiological conditions, LKB1 is primarily localized in the nucleus. However, under certain conditions, LKB1 is phosphorylated at Ser428 (pLKB1) and then exported to the cytoplasm, eventually inducing AMPK activation.^7^ LKB1‐AMPK can suppress mTORC1, and pharmacological inhibition of mTORC1 can alleviate pressure overload‐ and Ang II‐induced pathological myocardial hypertrophy and heart failure.^8^, ^9^

Nur77 (NR4A1), also known as TR3, is a member of the nuclear receptor superfamily that functions as an orphan receptor.^10^ It is classified as an immediate–early response gene due to its ability to be induced by various stimuli, including growth factors, inflammatory factors and stress, in different cell types.^11^ Nur77 possesses multiple biological functions, including the modulation of cell proliferation,^12^, ^13^ differentiation, development, apoptosis,^14^ metabolism and immunity.^15^ Recently, studies have reported that Nur77 modulates liver kinase B1 (LKB1) by binding and sequestering it in the nucleus, leading to the attenuation of AMP‐activated protein kinase (AMPK) activation. However, the function of Nur77 in this regard can be inhibited by certain compounds, such as ethyl (2,3,4‐trimethoxy‐6‐octanoylphenyl) acetate (TMPA),^16^ which interact with Nur77 at specific sites, leading to the release and nuclear export of LKB1 to the cytoplasm.^16^, ^17^ This event, in turn, mediates the phosphorylation of AMPK. The results of these studies have established both the transcriptional and post‐transcriptional links between Nur77 and LKB1.

Honokiol (HNK), a bioactive constituent obtained from Magnolia officinalis bark, has been frequently employed in traditional Chinese medicine. Studies in recent times have revealed its efficacy in demonstrating diverse therapeutic properties such as antioxidative, anti‐inflammatory, anti‐tumour and cardio‐protective effects.^18^, ^19^, ^20^, ^21^, ^22^, ^23^ Notably, HNK has been shown to mitigate hepatic triglyceride and lipogenic protein levels and attenuate fat deposition in high‐fat diet‐fed mice through a significant increase in LKB1 and AMPK phosphorylation.^24^ In relation to cancer, HNK has been found to curb breast tumorigenesis in mouse models by engaging in LKB1‐dependent pathways.^25^ Collectively, these findings suggest the crucial role of LKB1‐AMPK signalling in mediating HNK's cytoprotective effects. However, the precise mechanism underlying the modulation of LKB1‐AMPK signalling by HNK still remains elusive.

The present study investigated the effect of HNK on Ang II‐induced myocardial hypertrophy both *in vivo* and *in vitro*. Our findings reveal that HNK modulates the LKB1‐AMPK‐p70S6K signalling pathway, leading to the attenuation of myocardial hypertrophy. Further, we observed that LKB1‐mediated AMPK phosphorylation in the cytoplasm was suppressed by the binding of Nur77, which sequestered LKB1 in the nucleus. Notably, HNK disrupted the Nur77–LKB1 interaction, thereby promoting LKB1's nuclear export and facilitating AMPK phosphorylation. These results provide novel insights into the mechanism by which HNK mediates its cytoprotective effects through the regulation of the LKB1‐AMPK signalling pathway.

## 2 MATERIALS AND METHODS

### 2.1 Materials and reagents

Sigma‐Aldrich provided HNK, Ang II and DAPI, while WZ Biosciences (Shandong, China) supplied VigeneFection transfection reagent. Antibodies against LKB1, p‐LKB1, p70S6K, p‐p70S6K, AMPKα, p‐AMPKα and LaminB1 were obtained from Cell Signalling Technology, and anti‐β‐actin antibody was obtained from Santa Cruz Biotechnology. Abcam supplied antibodies against Nur77. Thermo Fisher Scientific provided anti‐rabbit and anti‐mouse secondary antibodies conjugated to HRP. These materials were utilized in the present study.

### 2.2 Animals

Animal experiments were carried out in accordance with the guidelines and regulations set forth by the US National Institutes of Health's ‘Guide for the Care and Use of Laboratory Animals’ and approved by the Animal Welfare and Policy Committee of Fujian Medical University (approval number: 2019‐0085). The experiments were also compliant with the ARRIVE (Animal Research: Reporting of *In Vivo* Experiments) guidelines. Eight‐week‐old male Sprague–Dawley rats (180 ± 20 g) were procured from the Shanghai Laboratory Animal Center, Chinese Academy of Sciences and kept in a specific pathogen‐free environment. The animals were randomly allocated into experimental groups using the random number generator function of SPSS version 24. All rats were euthanized by carbon dioxide. Investigators who were blinded to the treatment groups performed the statistical analysis and evaluated the results of all rats. All animal experimentation was conducted at the Central Laboratory of the First Affiliated Hospital of Fujian Medical University.

### 2.3 Animal model and experimental design

Cardiac remodelling was induced in the experimental animals by chronic infusion of Ang II (Sigma‐Aldrich) at a dose of 520 ng·kg^−1^·min^−1^ for a duration of 4 weeks, using ALZET® mini‐osmotic pumps (model number: 2006, DURECT Corporation), according to the manufacturer's guidelines.^26^ The control group received subcutaneous infusion of phosphate‐buffered saline (PBS). The rats were anaesthetised with a mixture of 2.0% isoflurane and oxygen (100%, airflow velocity: 1 L·min^−1^), and an incision was made in the skin on their backs to implant the capsule pump into the subcutaneous tissue. After suturing, the rats were returned to their housing cages and provided individualized feed till the completion of the experiment.

The animals were assigned randomly to one of the five experimental groups, including control (CTL), control group treated with HNK (2.5 mg·kg^−1^·d^−1^; CTL + HNK), Ang II‐treated group (520 ng·kg^−1^·min^−1^; Ang II), Ang II plus low‐dose HNK‐treated group (2.5 mg·kg^−1^·d^−1^; Ang II + HNK 2.5) and Ang II plus high‐dose HNK‐treated group (5 mg·kg^−1^·d^−1^; Ang II + HNK 5). HNK was dissolved in peanut oil and administered intraperitoneally twice daily. The high dose of HNK (5 mg·kg^−1^·d^−1^) was determined based on the literature^27^ and our pre‐experimental results. Blood pressure was measured weekly using the Softron BP‐2010A system (Softron Beijing Biotechnology Co. Ltd.) via the tail‐cuff method. The BP data were obtained by taking three measurements in the resting state, and the average value was calculated.^28^ All rats were subjected to environmental adaptation before the tail‐cuff measurements.

### 2.4 Routine echocardiogram and quantitative tissue velocity imaging (QTVI)

All rats were anaesthetised with 2% isoflurane through a facial mask. Transthoracic echocardiography using a 12S probe with frequencies ranging from 4 to 12 MHz was then performed. The M‐mode echocardiographic images of the parasternal left ventricle in the short‐axis view were recorded. Pulsed‐wave Doppler blood flow images of the apical four‐chamber view at the mitral level and Doppler tissue images (DTI) of the lateral and septal mitral annulus were also captured. Heart rates were synchronously measured using electrocardiography. Image quantification and analysis were done using the EchoPAC 202 Image Analysis System by GE Healthcare. From the parasternal left ventricular long‐axis view, the diastolic inner diameter of the aortic sinus and systolic anterior and posterior diameters of the left atrium were calculated. Using M‐mode echocardiography in the short‐axis view, the end‐diastolic left ventricular (LV) posterior wall thickness (LVPWD), end‐diastolic interventricular septum thickness (IVSD) as well as end‐systolic and end‐diastolic LV internal diameters (LVIDS and LVIDD, respectively) were measured at the LV papillary muscle level. The LV fractional shortening (LVFS) and LV ejection fraction (LVEF) were calculated using equations LVFS = [(LVIDD–LVIDS)/LVIDD] × 100 and LVEF = [(LV end‐diastolic volume − LV end‐systolic volume)/LV end‐diastolic volume] × 100, respectively. The relative wall thickness (RWT) was also calculated using the equation RWT = 2 × LVPWD/LVIDD.

Mitral orifice flow spectra and tissue Doppler dynamic images of the mitral annulus in apical four‐chamber cardiac view were obtained. The peak early diastolic velocity (PVE) was measured from the mitral orifice flow spectrum. To attain better temporal and velocity resolution, the Q‐analyze software (GE Healthcare) was employed for measuring tissue motion velocities of the mitral annulus on dynamic DTI. Specifically, peak velocities of tissue motion spectra of the lateral (s) and interstitial (s') septal mitral annular walls during systole were measured, and their mean (s ¯) was calculated. Similarly, peak velocities of tissue motion in the lateral (e) and interstitial (e’) walls of the mitral annulus during early diastole were measured, and the mean (ⅇ ¯) was calculated. Finally, the ratio PVE/s ¯ was calculated.

### 2.5 Analysis of heart tissue and blood serum

At the end of the fourth week, the animals were weighed. Following euthanasia by carbon dioxide, blood samples were obtained, and the heart was quickly excised and prepared for histology, protein and RNA extraction. The ratios of heart weight (HW) to tibia length (TBL) and left ventricular weight (LVW) to TBL were determined. The rat hearts were fixed in 10% formalin, dehydrated, paraffin‐embedded and cut into 5 mm sections. For histological evaluation, sections were collected on gelatin‐coated slides and stained with haematoxylin and eosin (H&E, Sigma‐Aldrich) and Picrosirius Red (PSR; Solarbio). Images of three hearts from each group were acquired using an Eclipse Ti microscope (Nikon) with a 20× objective. Only myocytes cut along the short axis with a visible nucleus were included in the analysis (a minimum of 200 cells per heart).^29^ The cell borders were manually delineated by a blinded operator. The two‐dimensional cross‐sectional areas were calculated using NIS‐Elements software (Nikon). For collagen analysis in the myocardial tissue, five fields of view were randomly chosen from each slide. For collagen volume fraction (CVF) analysis in the border area, six separate views (×400) were chosen, and the following equation was used to measure CVF: CVF = collagen area/total area.

Serum aspartate aminotransferase (AST) and alanine aminotransferase (ALT) levels were monitored continuously to assess liver function. Blood glucose, urea nitrogen, lipid and creatinine levels were measured using enzymatic and urease colorimetric assays. Furthermore, the concentrations of creatine kinase (CK), CK‐MB, lactic dehydrogenase and uric acid were determined by employing a colorimetric method provided by Roche. The serum levels of natriuretic peptide A, natriuretic peptide B, galectin‐3 (Gal‐3) and Ang II were analysed by employing an ELISA kit (Cloud‐Clone Corp).

### 2.6 Detecting of capillary and arteriolar density

For capillary and arteriolar density analysis, six heart cross sections were taken from each group of experimental animals and stained for CD31 (28083‐1‐AP, Proteintech) and IB4 (L2895, Sigma‐Aldrich), and the ratios of CD31 and IB4 positive areas to the total area were detected, respectively.

### 2.7 TUNEL assay

For apoptosis analysis, 5‐μm paraffin‐embedded heart sections were subjected to TUNEL staining using the TUNEL assay kit (11684795910, Roche) following the manufacturer's protocol. Six to eight fields were randomly selected from the heart of rats, for each section, under an optical microscope. The percentage of apoptotic cells was calculated as TUNEL‐positive nuclei number divided by the total number of nuclei identified by haematoxylin staining.

### 2.8 Cell culture

Neonatal rat ventricular myocytes (NRVMs) were isolated and cultured according to previously described methods.^30^, ^31^ In brief, the ventricles of newborn rats were minced, and the cells were isolated by digestion with type‐II collagenase (150 U·mL^−1^; Gibco) and stirring at 37°C. The isolated cardiomyocytes were seeded on 0.1% gelatin‐coated culture dishes (Merck) at a density of 7.5 × 10^4^ cells·cm^−2^ to obtain a confluent monolayer of spontaneously contracting cells after 24 h. Mitomycin C (10 μg·mL^−1^; Sigma‐Aldrich) was used for 4 h to inhibit cardiac fibroblast proliferation after seeding. The culture medium consisted of Dulbecco's modified Eagle's medium with 25 mM glucose, 5% fetal bovine serum, GlutaMAX™ and HEPES (all from Gibco). Cardiomyocyte purity was determined by immunofluorescence using α‐actinin antibody (Sigma‐Aldrich), and NRVMs with a purity of over 90% were used. HEK293T cells from the American Type Culture Collection were cultured in Dulbecco's modified Eagle's medium supplemented with 10% fetal bovine serum, 100 U·mL^−1^ penicillin and 100 μg·mL^−1^ streptomycin and maintained in a 5% CO2 atmosphere at 37°C. Empty adenovirus (Ad‐empty) or adenoviruses carrying cDNA of rat Nur77 or dominant negative Nur77 were applied to freshly isolated and cultured cardiomyocytes at a multiplicity of infection (MOI) of 100 for 40 h prior to 30 min application of HNK.

### 2.9 Construction of plasmids

The present study employed PCR to generate Nur77 cDNA, using forward primer (5′‐ACT GGA TAC ACC CGT GAC CT‐3′) and reverse primer (5′‐ACA GGG CAG TCC TTG TTA GG‐3′). The amplified products were cloned into the pENTER vector containing a cytomegalovirus (CMV) promoter and C‐terminal FLAG and His tags (Vigene Biosciences, Jinan, China). The resulting pENTER‐CMV‐Nur77 expression vector was confirmed by sequencing. HEK293T cells were transfected with 1 μg of pENTER‐CMV‐Nur77 expression vector or pENTER‐CMV control vector, using 3 μL of VigeneFection transfection reagent.

### 2.10 Subcellular fractionation

Nuclear and cytosolic fractions were harvested using NE‐PER™ Nuclear and Cytoplasmic Extraction Reagents (Thermo Fisher Scientific), according to the manufacturer's instructions.

### 2.11 Real‐time reverse transcription‐PCR

First‐strand cDNA was synthesized using the RevertAid First Strand cDNA Synthesis Kit (Thermo Fisher Scientific). Amplification of the Nur77, LKB1, and β‐actin genes was performed using specific primers. The Nur77 gene was amplified using the forward primer 5′‐ACT GGA TAC ACC CGT GAC CT‐3′ and reverse primer 5′‐ACA GGG CAG TCC TTG TTA GG‐3′. The LKB1 gene was amplified using the forward primer 5′‐TTG TTT GAC ATT GAG GAC GGC‐3′ and reverse primer 5′‐GTG CCA TTC ACA CAA ACA GCC‐3′. The β‐actin gene was amplified using the forward primer 5′‐TGG TGA AGG TCG GTG TGA AC‐3′ and reverse primer 5′‐GAA TTT GCC GTG AGT GGA GTC‐3′.

### 2.12 Western blot, co‐immunoprecipitation and immunofluorescence assays

Western blot, co‐immunoprecipitation and immunofluorescence assays were conducted according to established protocols.^32^, ^33^ For western blot, cellular proteins ranging from 25 to 50 μg were separated via SDS‐PAGE and transferred to PVDF membranes. The membranes were blocked with 5% skim milk in TBST buffer (150 mM NaCl, 0.1% Tween‐20 and 50 mM Tris–HCl, pH 7.4) and subsequently incubated overnight with primary antibodies, followed by detection with secondary antibodies. Detection of the final immunoreactive products was performed using an Invitrogen iBright™ FL1500 chemiluminescence system (ThermoFisher Scientific). Co‐immunoprecipitation assay was conducted using the Dynabeads™ Co‐Immunoprecipitation Kit (ThermoFisher Scientific), following the manufacturer's instructions.

The immunofluorescence analysis was conducted by culturing cells on glass slides for 24 h, and subjecting them to experimental treatment. The cells were then permeabilized with PBS containing 0.05% Triton™ X‐100 and 0.1 M glycine on ice for 20 min and subsequently blocked with 1% BSA in PBS for 30 min at 18–25°C. Mouse anti‐Nur77 antibody (1:100) and rabbit anti‐LKB1 antibody (1:100) were added to the cells and incubated at 4°C for 12 h, followed by detection using an anti‐mouse immunoglobulin G (IgG) conjugated with Alexa Fluor® Plus 594 (1:200) and anti‐rabbit IgG conjugated with Alexa Fluor® Plus 488 (1:200, all from Thermo Fisher Scientific), respectively. Images were acquired with an LSM‐510 confocal laser‐scanning microscope (Carl Zeiss).

### 2.13 Statistical analysis

The data are presented as the mean ± SEM. Statistical analysis was conducted using one‐way analysis of variance (anova) followed by Tukey's post‐hoc test if the data were normally distributed. Additionally, the post‐hoc test was conducted only if the F‐statistic was significant (*p* < 0.05). For non‐normally distributed data, the Kruskal–Wallis H test was used, and individual mean comparisons were performed using the Mann–Whitney *U* test. The statistical analyses were conducted using SPSS version 16, and the significance level was set at *p* < 0.05.

## 3 RESULTS

### 3.1 HNK eliorates Ang II‐induced myocardial hypertrophic remodelling and diastolic dysfunction

**FIGURE 1:**
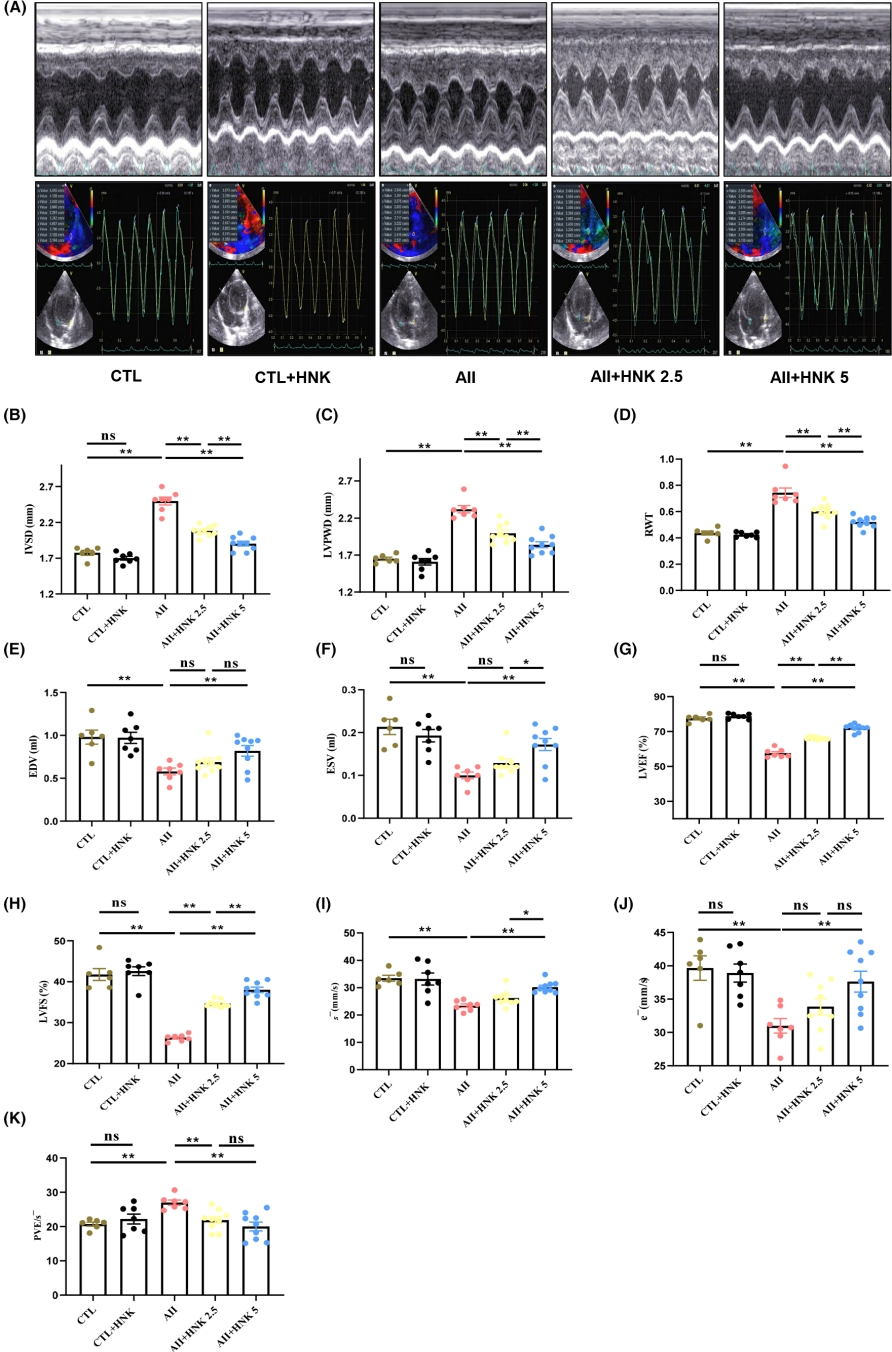
HNK ameliorates Ang II‐induced myocardial hypertrophic remodelling and diastolic dysfunction. (A) Representative M‐model echocardiograms and Doppler recordings at 4 weeks after Ang II infusion. (B–D) Quantitative analysis of IVSD, relative wall thickness (RWT) and left ventricular posterior wall thickness (LVPWD) (*n* = 6–9 rats per group). (E–H) Echocardiography measurements of LV end‐diastolic volume (EDV), LV end‐systolic volume (ESV), left ventricular ejection fraction (LVEF) and left ventricular fractional shortening (LVFS) (*n* = 6–9 rats per group), at 4 weeks after Ang II infusion. (I) $\bar{s}$, mean of peak velocity of the lateral wall and interval septum of the mitral annulus during systole. (J) $\bar{\mathrm{e}}$, mean of peak velocities in the lateral and interstitial walls of the mitral annulus during early diastole. (K) The PVE is the peak flow rate at the orifice of the mitral valve. ns indicates no statistical significance. *p < 0.05 and **p < 0.01, as assessed using one‐way anova followed by Tukey's multiple comparison test. Data have been presented as mean ± S.E.M.

In this study, the effect of HNK on myocardial dysfunction and remodelling induced by continuous Ang II infusion was investigated using a rat model. Our findings indicated that Ang II administration induced cardiac hypertrophy and dysfunction, as evidenced by elevated IVSD, LVPWD, RWT, HW, LVW values, as well as HW/TL and LVW/TL ratios (Figure 1A–C) and Figure S1A–D), along with decreased EDV, ESV, LVEF, LVFS, and LVDD, LVDS (Figure 1E–H), Figure S1E,F and Table 1). Moreover, s ¯ and ⅇ ¯ decreased (Figure 1I,J), while the PVE/s ¯ ratio increased in the Ang II‐induced group (Figure 1K), as detected by QTVI technology. However, HNK treatment reversed these changes. In addition, Ang II infusion increased serum Ang II levels, as well as diastolic and systolic BP levels in rats, while HNK treatment did not alter serum Ang II levels or Ang II‐induced BP (Tables 1 and 2) and Figure S1G,H). No changes in body weight were observed in any experimental groups. In summary, our results suggest that HNK can ameliorate myocardial remodelling and function in Ang II‐infused rats, without altering BP.

**TABLE 1:** Physical and conventional echocardiographic parameters in SD rats and Ang II‐infused rats treated with vehicle or HNK.

Parameters	CTL	CTL+HNK	AII	AII+HNK 2.5	AII+HNK 5
BW (g)	362.17 ± 12.67	361.83 ± 7.94	317.43 ± 15.95	284.56 ± 20.11	306.44 ± 40.68
SBP (mmHg)	126.1 ± 2.59	125.7 ± 1.1	203.14 ± 13.27**	204.99 ± 34.63	204.92 ± 24.24
DBP (mmHg)	103.35 ± 2.22	103.85 ± 1.21	163.19 ± 19.76**	168.44 ± 27.12	172.01 ± 20.75
HW (g)	1.0 ± 0.08	0.99 ± 0.07	1.31 ± 0.07**	1.11 ± 0.11^##^	1.09 ± 0.14^##^
LVW (g)	0.71 ± 0.07	0.71 ± 0.04	0.98 ± 0.05**	0.84 ± 0.09^##^	0.81 ± 0.1^##^
HW/TL (g/cm)	0.25 ± 0.02	0.24 ± 0.01	0.32 ± 0.01**	0.27 ± 0.03^##^	0.27 ± 0.03^##^
LVW/TL (g/cm)	0.18 ± 0.02	0.18 ± 0.01	0.24 ± 0.01**	0.21 ± 0.02^##^	0.20 ± 0.02^##^
AOD (mm)	3.97 ± 0.32	3.72 ± 0.25	4.19 ± 0.23	4.11 ± 0.16	3.95 ± 0.30
LAD (mm)	4.66 ± 0.39	4.67 ± 0.32	4.91 ± 0.38	4.47 ± 0.20	4.67 ± 0.51
IVSD (mm)	1.78 ± 0.08	1.7 ± 0.07	2.50 ± 0.14**	2.08 ± 0.07^##^	1.9 ± 0.10^##&&^
RWT	0.44 ± 0.04	0.42 ± 0.02	0.74 ± 0.09**	0.6 ± 0.06^##^	0.52 ± 0.04^##&&^
LVPWD (mm)	1.65 ± 0.05	1.61 ± 0.12	2.32 ± 0.13**	1.99 ± 0.13^##^	1.84 ± 0.12^##&&^
LVDD (mm)	7.59 ± 0.58	7.58 ± 0.49	6.28 ± 0.42**	6.67 ± 0.46	7.10 ± 0.62^##^
LVDS(mm)	4.41 ± 0.34	4.24 ± 0.32	3.35 ± 0.21**	3.69 ± 0.28^#^	4.07 ± 0.39^##&^
EDV (mL)	0.98 ± 0.2	0.97 ± 0.17	0.58 ± 0.10**	0.69 ± 0.14	0.82 ± 0.18^##^
ESV (mL)	0.21 ± 0.04	0.19 ± 0.04	0.10 ± 0.02**	0.13 ± 0.03	0.17 ± 0.04^##&^
LVFS (%)	41.78 ± 3.6	42.59 ± 2.84	26.32 ± 0.85**	34.64 ± 0.88^##^	38.02 ± 1.97^##&^
LVEF (%)	77.55 ± 1.99	78.78 ± 1.52	57.6 ± 2.45**	66.14 ± 0.77^##^	72.02 ± 2.14^##&&^
HR (beats/min)	390.85 ± 23.5	376.74 ± 46.78	409.01 ± 26.04	410.04 ± 41.02	382.05 ± 57.34
PVE (mm/s)	819.25 ± 91.21	854.68 ± 101.74	831 ± 27.15	742.74 ± 138.8	740.02 ± 89.33
$\bar{s}$ (mm/s)	33.47 ± 2.68	33.18 ± 5.82	23.41 ± 1.81**	26.21 ± 2.9	30.21 ± 2.13^##&^
*ē* (mm/s)	39.67 ± 4.5	38.91 ± 3.56	30.98 ± 2.9**	33.87 ± 3.68	37.63 ± 4.71^##^
PVE/$\bar{s}$	20.71 ± 1.46	22.2 ± 3.86	26.97 ± 1.99**	21.87 ± 3.01^##^	20.02 ± 3.9^##^

*Note*: Values have been presented as mean ± SEM (*n* = 6–9 rats per group).

Abbreviations: $\bar{e}$, mean of peak velocities in the lateral and interstitial walls of the mitral annulus during early diastole; $\bar{s}$, mean of peak velocity of the lateral wall and the interval septum of the mitral annulus during systole; Ang II, angiotensin II; AOD, aorta internal diameter; CTL, control; DBP, diastolic blood pressure; EDV, end‐diastolic volume; ESV, end‐systolic volume; NK, honokiol; IVSD, interventricular septal thickness; LAD, left atrial internal diameter; LVDD, left ventricular end‐diastolic dimension; LVDS, left ventricular end‐systolic dimension; LVEF, left ventricular ejection fraction; LVFS, left ventricular fractional shortening; LVPWD, left ventricular posterior wall thick; PVE, peak flow rate at the mitral valve orifice; RWT, relative wall thick; SBP, systolic blood pressure.

**p < 0.01, relative to CTL group; ^#^ *p* < 0.05 and ^##^ *p* < 0.01, relative to the Ang II group; ^&^ *p* < 0.05 and ^&&^ *p* < 0.01, relative to the Ang II±HNK 2.5 group.

**TABLE 2:** Blood parameters of the Ang II‐infused rats treated with vehicle or HNK for 4 weeks.

Parameter	CTL	CTL + HNK	AII	AII + HNK 2.5	AII + HNK 5
GLU (mM)	6.21 ± 0.41	6.27 ± 0.33	6.57 ± 0.43	6.62 ± 0.47	6.46 ± 0.54
ALT (U/L)	14.00 ± 2.24	15.00 ± 4.14	16.24 ± 5.40	17.14 ± 5.28	16.52 ± 4.62
AST (U/L)	26.48 ± 5.12	27.24 ± 4.42	28.24 ± 4.24	27.47 ± 5.28	28.74 ± 6.16
BUN (mM)	5.86 ± 0.49	5.72 ± 0.64	5.79 ± 0.83	5.65 ± 0.88	5.78 ± 0.74
SCR(μ M)	15.81 ± 1.58	14.94 ± 2.14	16.14 ± 2.81	16.96 ± 1.89	14.57 ± 2.10
CK (U/L)	212.52 ± 7.24	217.52 ± 6.48	215.14 ± 8.82	214.28 ± 9.04	216.52 ± 8.18
CK‐MB (U/L)	64.2 ± 3.62	65.2 ± 4.63	66.8 ± 6.41	68.2 ± 8.5	66.94 ± 6.25
LDH (U/L)	1782 ± 116.73	1812 ± 162.48	1858 ± 136.86	1740 ± 187.87	1897 ± 192.48
UA (μ M)	6.43 ± 2.15	6.92 ± 1.53	6.5 ± 1.26	12.04 ± 1.79**	19.66 ± 6.87**#
TC (mM)	1.71 ± 0.12	1.68 ± 0.35	2.25 ± 0.2**	1.82 ± 0.39^##^	1.52 ± 0.25^##&&^
TG (mM)	1.25 ± 0.33	1.27 ± 0.46	1.28 ± 0.41	1.14 ± 0.39	0.79 ± 0.26**
LDL‐C (mM)	0.35 ± 0.07	0.36 ± 0.12	0.36 ± 0.10	0.41 ± 0.09	0.35 ± 0.10
HDL‐C (mM)	0.45 ± 0.06	0.46 ± 0.12	0.49 ± 0.13	0.44 ± 0.06	0.42 ± 0.09
AII (pg/ml)	138.93 ± 16.68	135.16 ± 13.49	165.16 ± 18.38**	159.33 ± 22.4	161.75 ± 20.01
ANP (pg/ml)	411.22 ± 57.85	427.73 ± 23.6	703.89 ± 29.19**	525.64 ± 20.66**##	447.63 ± 25.13^##&&^
BNP (pg/ml)	304.14 ± 40.61	295.84 ± 42.93	578.42 ± 42.48**	431.73 ± 47.22^**##^	321.29 ± 38.27^##&&^

*Note*: Values are presented as mean ± SEM (*n* = 6–9 rats per group).

Abbreviations: AII, Angiotensin II; ALT, alanine transaminase; ANP, atrial natriuretic peptide; AST, aspartate transaminase; BNP, brain natriuretic peptide; BUN, blood urea nitrogen; CK, creatine kinase; CK‐MB, creatine kinase isoenzymes; CTL, Control; GLU, indicates glucose; HDL‐C, high‐density lipoprotein cholesterol; HNK, Honokiol; LDH, lactate dehydrogenase; LDL‐C, low‐density lipoprotein cholesterol; SCR, serum creatinine; TC, total cholesterol; TG, triglyceride; UA, uric acid.

^**^ *p* < 0.01 relative to CTL group; ^#^ *p* < 0.05 or ^##^ *p* < 0.01 relative to AII group; ^&&^ *p* < 0.01 relative to AII + HNK 2.5 group.

### 3.2 HNK improves lipid metabolism but increases serum uric acid

Our investigation of Ang II‐infused rats revealed significant elevations in total cholesterol (TC), atrial natriuretic peptide (ANP), and brain natriuretic peptide (BNP) levels compared to the control group (Table 2) and Figure S2A–C). Following low‐ or high‐dose HNK treatment for 4 weeks, we observed a marked reduction in TC, ANP, BNP and triglyceride (TG) levels in Ang II‐infused rats (Table 2) and Figure S2A–D). Nevertheless, the administration of HNK increased serum uric acid levels in a dose‐dependent manner (Table 2) and Figure S2E). We did not observe any signs of liver, skeletal muscle or kidney toxicity associated with HNK treatment (Table 2).

### 3.3 HNK inhibits cardiomyocyte hypertrophy *in vivo* and *in vitro*

**FIGURE 2:**
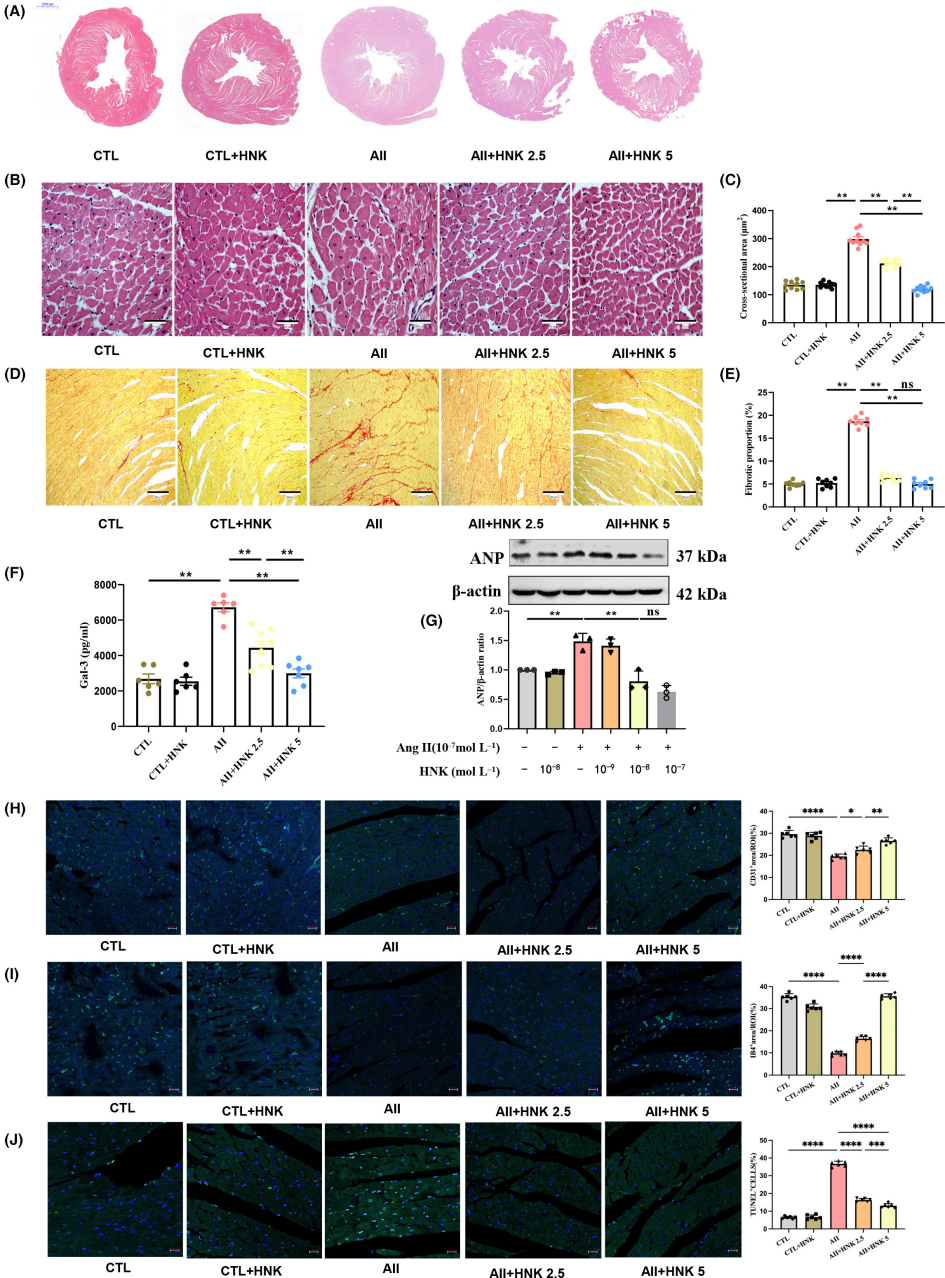
HNK inhibits cardiomyocyte hypertrophy *in vivo* and *in vitro*. (A) Representative LV sections stained with haematoxylin and eosin (H&E). Scale bar: 2000 μm. (B–E) Representative LV sections stained with H&E and Sirius red (SR) to evaluate cardiomyocyte hypertrophy (B) and fibrosis (D), respectively. Scale bar: 50 or 200 μm. (C) Quantification of the cardiomyocyte cross‐sectional area in the H&E‐stained sections. (E) Quantification of collagen in the SR‐stained cardiomyocytes. (F) Levels of galectine‐3 (Gal‐3) at the end of the study, as measured using ELISA assay. ns indicates no statistical significance. (G) Representative immunoblots and quantification of ANP protein upon treatment of NRVMs with Ang II (10^−7^ mol⋅L^−1^) for 24 h (*n* = 3). (H,I) Representative confocal IF images of CD31 and IB4 obtained from capillaries and arteries in LV and the quantification of the positive area of ORI. (J) Representative LV sections stained with TUNEL.DAPI staining denotes nuclei; myocardial apoptosis index measured as the positive cell divided by total cell. Data have been shown as mean ± S.E.M. *n* = 6–9 rats per group; **p < 0.01, as assessed using one‐way anova followed by Tukey's multiple comparison test. CTL, control; AII, angiotensin II; HNK, honokiol.

Galactin‐3 (Gal‐3) has been implicated in fibrogenesis, inflammation, and myocardial remodelling. Previous studies have shown that repression of Gal‐3 can reverse left ventricular dysfunction induced by isoproterenol by attenuating myocardial inflammation and fibrogenesis.^34^ Three pro‐hypertrophy markers, namely natriuretic peptide A, natriuretic peptide B and myosin heavy chain beta, are commonly used to evaluate myocardial hypertrophy.^35^ In this study, we examined whether HNK could protect against Ang II‐induced myocardial hypertrophy in rats by performing H&E staining, SR staining, and Gal‐3 testing assays *in vivo*. The results showed that the Ang II‐infused rats exhibited thicker endocardium and larger cross‐sectional area of myocardial cells compared to the control rats (Figure 2A–C). Additionally, the SR staining showed increased and scattered red‐stained fibres in the myocardial tissue of the rats with 4 weeks of Ang II infusion (Figure 2D,E). Furthermore, the Gal‐3 levels were significantly increased in the Ang II‐infused group (Figure 2F). After 4 weeks of Ang II infusion, HNK was found to attenuate cardiac hypertrophy, fibrosis and serum Gal‐3 levels in a dose‐dependent manner (Figure 2F). Staining of CD31 and IB4 showed a bit increase in arteries and significant increase in capillaries with HNK + Ang II group (Figure 2H,I), Figure S3A,B. Meanwhile, a TUNEL assay was performed to detect cardiomyocyte apoptosis. The results showed that cardiomyocyte apoptosis can be attenuated after treating with HNK (Figure 2, Figure S3C). Furthermore, *in vitro* experiments demonstrated that HNK inhibited ANP protein levels in a concentration‐dependent manner, Ang II and HNK concentration as indicated (Figure 2G).

### 3.4 HNK alleviates cardiomyocyte hypertrophy via the LKB1/AMPK/p70S6K signalling pathway

**FIGURE 3:**
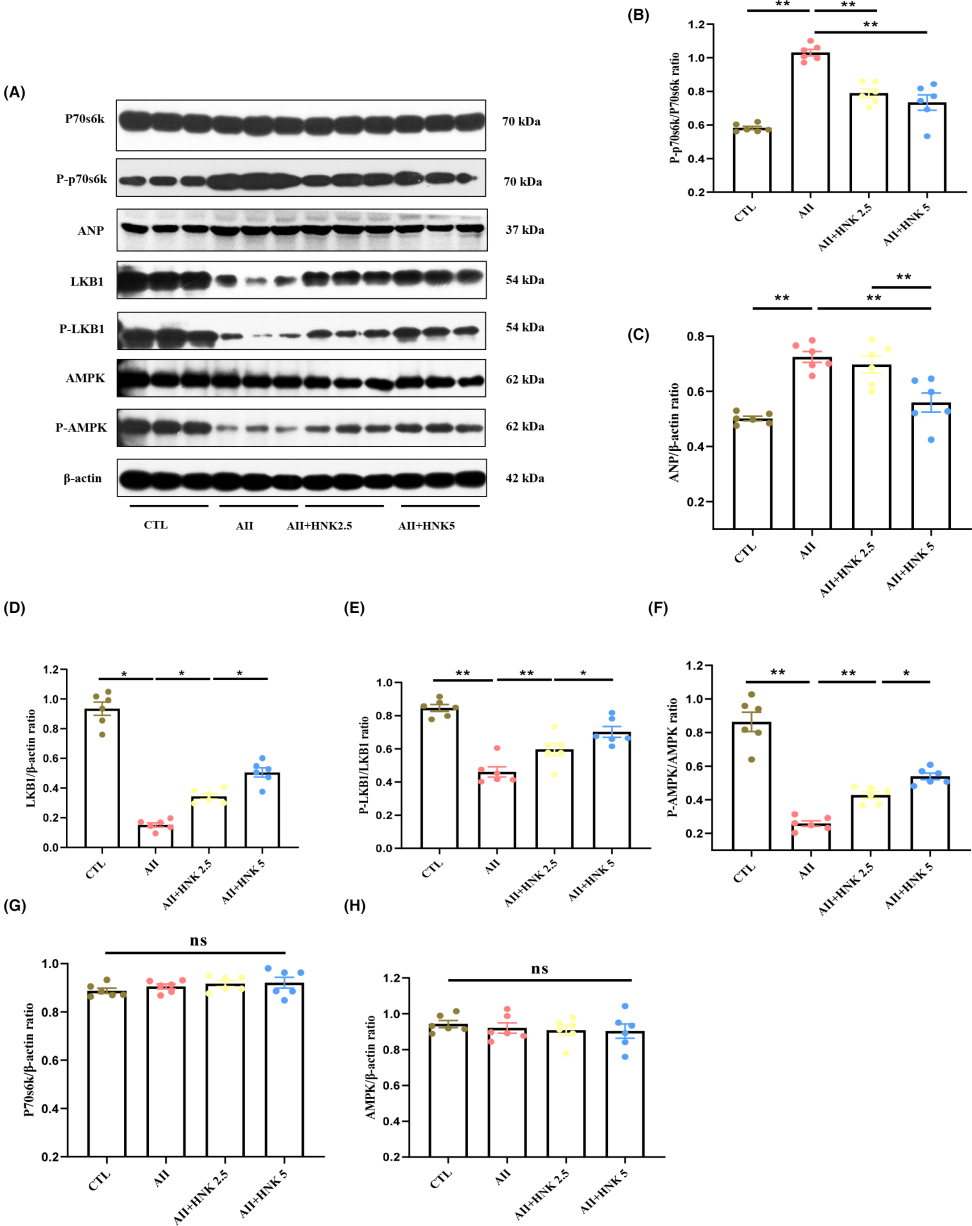
HNK alleviates cardiac hypertrophy via the LKB1/AMPK/p70S6K signalling pathway in AII‐infused rats. (A) Major proteins of the LKB1/AMPK/p70S6K signalling pathway were examined in myocardial tissues from CTL and cardiac hypertrophy rats, by means of immunoblotting, the results for which are quantified in (B‐G) (*n* = 6 rats per group). ns indicates no statistical significance. Data have been shown as mean ± S.E.M. *p < 0.05 and **p < 0.01, as assessed using one‐way anova followed by Tukey's multiple comparison test. CTL, control; AII, angiotensin II; HNK, honokiol.

**FIGURE 4:**
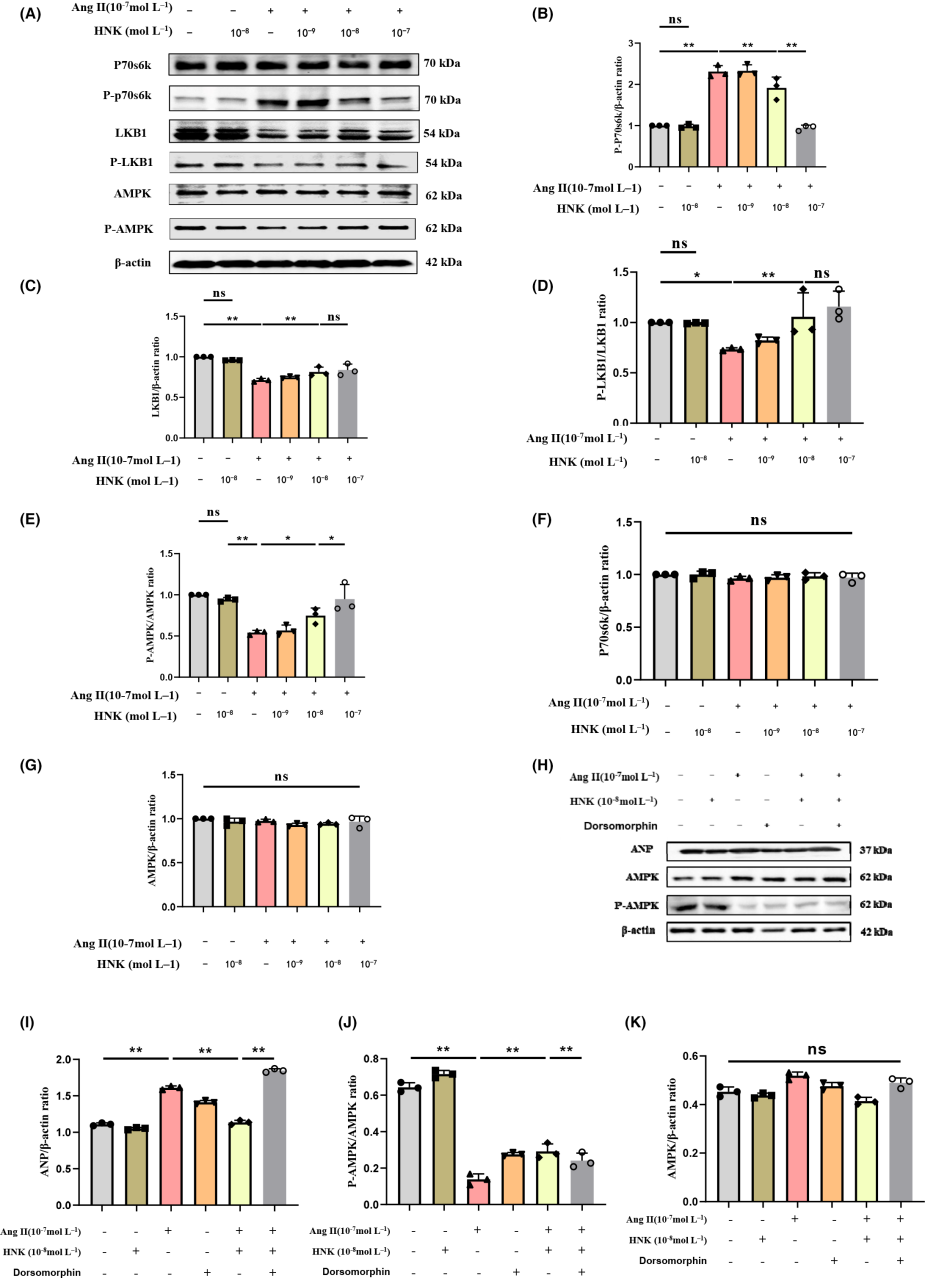
HNK antagonizes the regulatory effects of Ang II on the LKB1/AMPK/p70S6K pathway in NRVMs cultured *in vitro*. (A–G) Representative immunoblots and quantification of the major proteins of the LKB1/AMPK/p70S6K signalling pathway in NRVMs subjected to treatment with HNK (10^−9^ to 10^−7^ mol⋅mL^−1^) for 30 min, followed by activation with Ang II (10^−7^ mol⋅mL^−1^) for 1 h, to induce hypertrophy (*n* = 3). (H–K) Representative immunoblots and quantification of the major proteins of the LKB1/AMPK/p70S6K signalling pathway in NRVMs subjected to treatment with HNK (10^−8^ mol⋅mL^−1^) and Dorsomorphin (10^−5^ mol⋅mL^−1^) for 30 min, followed by activation with Ang II (10^−7^ mol⋅mL^−1^) for 1 h, to induce hypertrophy (*n* = 3). ns indicates no statistical significance. *p < 0.05 and **p < 0.01, as assessed using a one‐way analysis of variance followed by Tukey's multiple comparison test. Data have been shown as mean ± S.E.M.

In a previous study, we demonstrated that LKB1/AMPK/p70S6K signalling plays a pivotal role in the development of hypertension‐induced myocardial hypertrophy in rats.^36^ Through our *in vivo* experiments, we found that the myocardial levels of p‐p70S6K and ANP were significantly higher in Ang II‐infused rats than in the control group (Figure 3A–C), whereas the levels of LKB1, p‐LKB1 and p‐AMPK were markedly reduced in the Ang II‐infused group (Figure 3D–F). Notably, HNK treatment dose‐dependently reduced p‐p70S6K expression (Figure 3A–C) and increased the expression of LKB1, p‐LKB1 and p‐AMPK (Figure 3D–F). However, the protein levels of p70S6K and AMPK were comparable among the four groups (Figure 3G,H), and HNK treatment alone did not alter the expression of these molecules in healthy control rats (Figure S4A–I). In addition, we observed higher levels of p‐p70S6K and lower levels of LKB1, p‐LKB1 and p‐AMPK in NRVMs after 1 h of Ang II treatment compared to those in the control group (Figure 4A). HNK treatment concentration‐dependently reduced p‐p70S6K levels (Figure 4A,B) and increased LKB1, p‐LKB1 and p‐AMPK expression in NRVMs (Figure 4C–E), while the levels of p70S6K and AMPK proteins remained unchanged (Figure 4F,G). Meanwhile, to demonstrate the important role played by AMPK signalling in cardiomyocyte hypertrophy, we performed AMPK inhibition experiments in NRVMs, which showed a significant increase in ANP expression and significant hypertrophy of cardiomyocytes in the presence of Dorsomorphin, an AMPK inhibitor (Figure 4H–K). Nur77 knockdown was performed in order to proove that the effects on cardiomyocyte hypertrophy observed upon Ang II and HNK administration are really dependent on Nur77. The results indicated that the AMPK signalling pathway was activated after Nur77 knockdown (Figure S5A–G). Overall, our findings suggest that the LKB1/AMPK/p70S6K signalling pathway is implicated in the development of Ang II‐induced myocardial hypertrophy, and that HNK can attenuate cardiomyocyte hypertrophy by modulating this pathway.

### 3.5 HNK mediates the modulation of the LKB1/AMPK signalling pathway by Nur77

**FIGURE 5:**
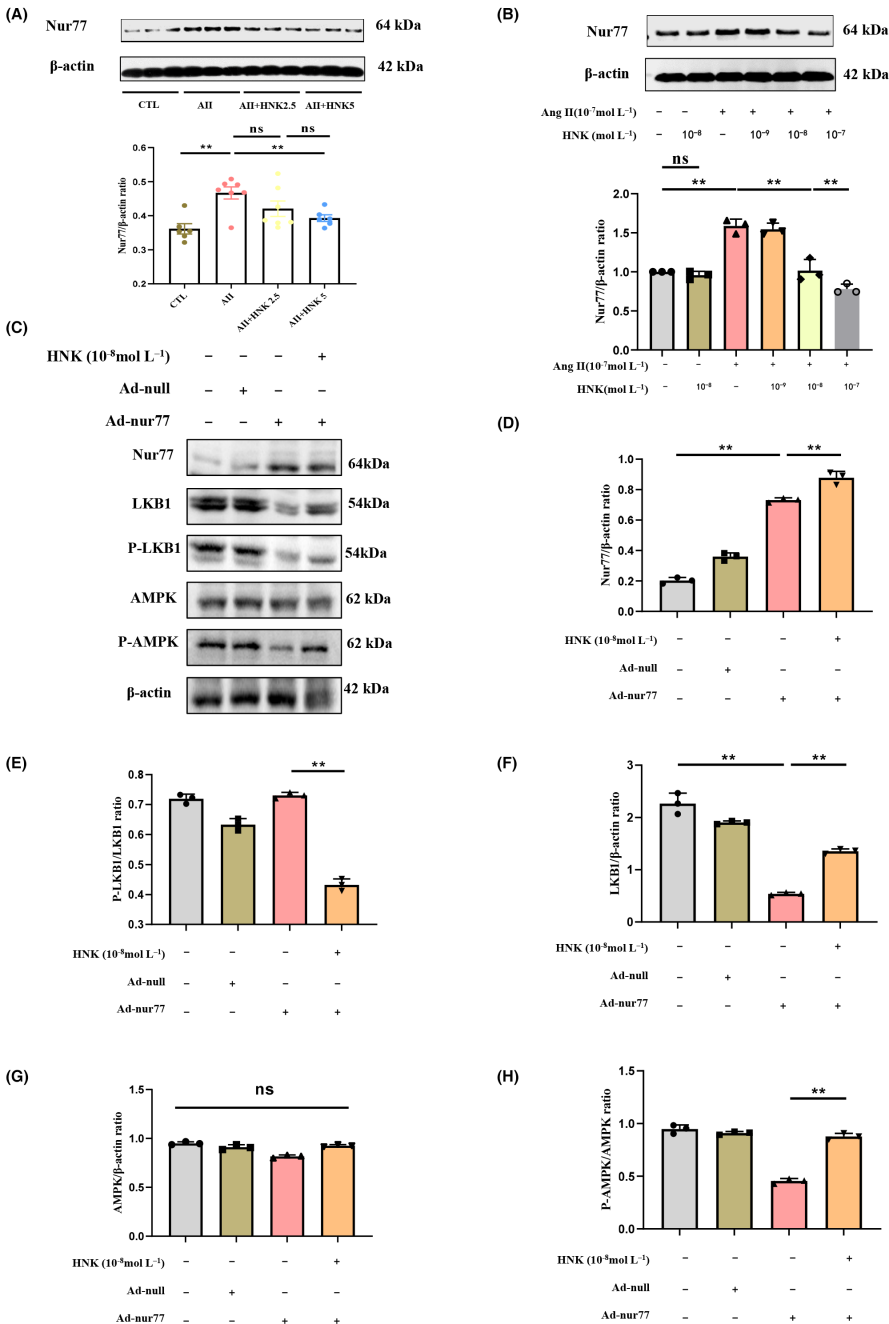
HNK mediates modulation of the LKB1/AMPK signalling pathway by Nur77. (A) Nur77 protein levels in the myocardial tissues from CTL and cardiac hypertrophy rats were examined by means of immunoblotting, following which the results were quantified (*n* = 6 rats per group). (B) Representative immunoblots and quantification of Nur77 protein levels in NRVMs treated as described in Figure 4. (C–H) NRVMs were treated with HNK for 30 min, following which they were transfected with Flag‐His‐tagged Nur77 plasmid using Lipofectamine™ 3000. HNK, honokiol; Ang II, angiotensin II; ns, no statistical significance. **p < 0.01, as assessed using one‐way anova followed by Tukey's multiple comparison test. Data have been shown as mean ± S.E.M.

Numerous studies have highlighted the essential roles of Nur77 in animals, including myocardial hypertrophy,^29^, ^37^ fibrosi ^[39]^ and apoptosis.^38^ Consistent with previous findings, our *in vivo* experiment showed that infusion of Ang II for 4 weeks significantly increased Nur77 expression (Figure 5A). *In vitro*, we also observed that Nur77 expression was upregulated in NRVMs stimulated with Ang II for 1 h (Figure 5B). Moreover, upon exploring the time‐effect relationship of Ang II intervention on Nur77 expression, we noted that Ang II induced a time‐dependent increase in Nur77 protein level, with the peak at 1 h of intervention (Figure S6A,B). Overexpression of Nur77 in 293T cells inhibited the LKB1/AMPK signalling, but treatment with HNK reversed this result (Figure S7A–G). Meanwhile, overexpression of Nur77 in NRVMs by adenoviral transfection showed that overexpression of Nur77 significantly inhibited the LKB1/AMPK signalling pathway, and that this inhibition could be reversed by HNK intervention (Figure 5C–H). Therefore, Nur77 is an upstream molecule of the LKB1/AMPK signalling pathway that may play a crucial role in modulating the LKB1/AMPK pathway by HNK.

### 3.6 HNK promotes dissociation of the Nur77–LKB1 complex to activate downstream signalling pathways

**FIGURE 6:**
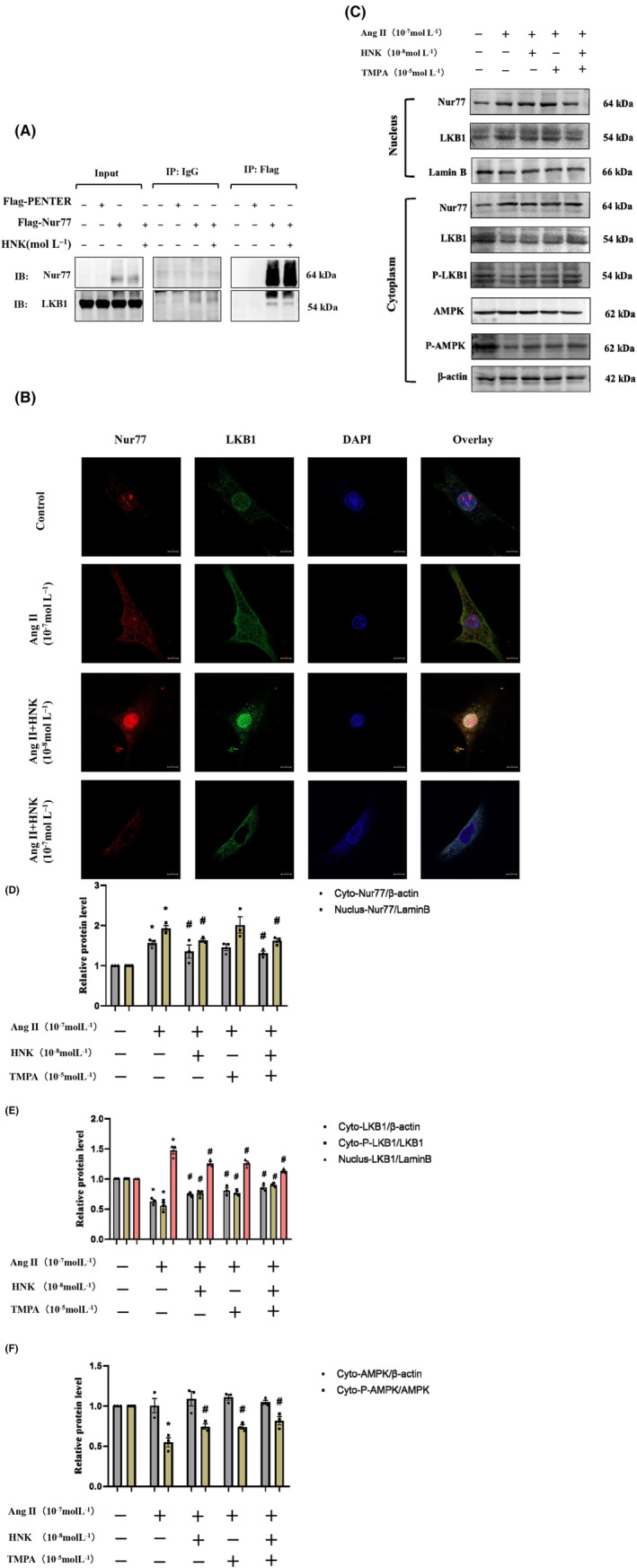
HNK promotes dissociation of the Nur77–LKB1 complex to activate downstream signalling pathways. (A) Co‐immunoprecipitation results for Nur77 and LKB1. Flag‐His‐tagged Nur77 plasmids were transfected to HEK293T cells incubated with or without HNK (10^−8^ mol L^−1^). The precipitate was analysed using a western blot, with anti‐Nur77 and anti‐LKB1 antibodies. (B) Subcellular co‐localization of Nur77 and LKB1. Immunofluorescence in the treated NRVMs was examined using confocal fluorescence microscopy. Scale bar: 10 μm (*n* = 3). (C–F) NRVMs were pre‐treated with HNK (10^−9^–10^−8^ mol⋅mL^−1^) and TMPA(10^−5^ mol L^−1^) for 30 min and then stimulated with Ang II (10^−7^ mol⋅mL^−1^) for 1 h, following which the expression levels of Nur77, total and phosphorylated LKB1 and AMPK were analysed in the cytosolic and nuclear proteins. Representative blots and quantification data are shown. HNK, honokiol; Ang II, angiotensin II. ns indicates no statistical significance. *p < 0.05 and **p < 0.01, as assessed using one‐way anova followed by Tukey's multiple comparison test. Data have been presented as mean ± S.E.M.

In the current study, we investigated the mechanism by which HNK counteracts Ang II‐induced cardiomyocyte hypertrophy. In the nucleus, LKB1 binds to Nur77 to form a complex^16^; TMPA promotes the dissociation of this complex, and thereby activates the downstream AMPK, to reduce lipid accumulation in HepG2 cells and mouse primary hepatocytes.^39^ Our immunoprecipitation experiments confirmed that Nur77 can bind to LKB1 in the nucleus (Figure 6A). Further experiments using immunofluorescence and nucleoplasm‐separation experiments showed that HNK reduced the expression of Nur77, attenuated the binding of Nur77 to LKB1, and activated the AMPK signalling pathway in a concentration‐dependent manner (Figure 6B,C,F) and Figure S8). In NRVMs, Ang II intervention increased the aggregation of Nur77 and LKB1 in the nucleus, while downregulating their expression in the cytoplasm, which led to more LKB1 sequestration in the nucleus and attenuated the downstream AMPK signalling cascade response. We have confirmed that HNK could block the formation of the Nur77–LKB1 complex and promote nuclear export of LKB1 to the cytoplasm and TMPA also have this effect, so we wanted to know if there was a synergistic effect between the two drugs. From the results, it is clear that there is no synergy between the two drugs (Figure 6C–E). Our findings suggest that HNK counteracts Ang II‐induced cardiomyocyte hypertrophy by promoting the dissociation of the Nur77–LKB1 complex.

### 3.7 HNK inhibits Nur77 transcription and promotes ubiquitous degradation of Nur77 protein

**FIGURE 7:**
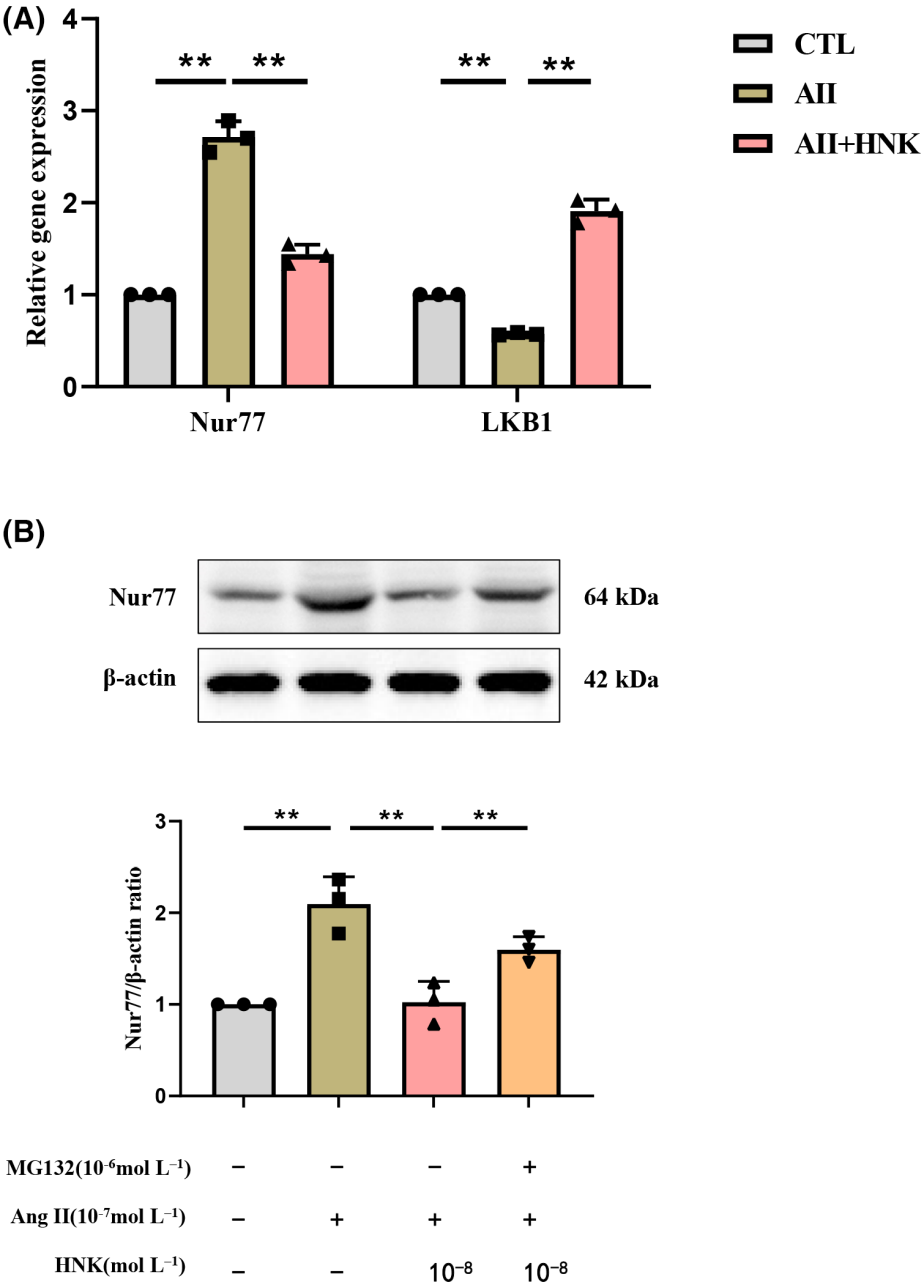
HNK inhibits transcription of Nur77 and promotes its ubiquitous degradation. (A) mRNA levels of Nur77 and LKB1 (*n* = 3) in NRVMs were examined using qPCR and have been represented as arbitrary units normalized to the values of the CTL group. (B) NRVMs were treated with HNK (10^−8^ mol⋅mL^−1^) for 30 min, followed by stimulation with Ang II (10^−7^ mol⋅mL^−1^) and MG132 (10^−6^ mol⋅mL^−1^) for 1 h. The average Nur77 protein expression was normalized to that of β‐actin. HNK, honokiol; Ang II, angiotensin II. ns, no statistical significance. *p < 0.05 and **p < 0.01, as assessed using one‐way anova followed by Tukey's multiple comparison test. Data have been shown as mean ± S.E.M.

The present study investigated the effects of HNK on Nur77 protein levels and AMPK signalling. The observed results revealed that HNK treatment alone has no effect on Nur77 expression but only reverses Ang II effect on Nur77. Therefore, we next checked Nur77 transcription and degradation after an HNK alone treatment to confirm that HNK inhibits transcription of Nur77 and promotes its ubiquitous degradation. Real‐time PCR experiments revealed that Ang II upregulated Nur77 mRNA levels while downregulating LKB1 mRNA levels in NRVMs. Notably, HNK administration significantly reversed the effects of Ang II on Nur77 and LKB1 mRNA levels (Figure 7A). To examine whether HNK promotes Nur77 degradation through the ubiquitin‐proteasome pathway, NRVMs were pre‐treated with the ubiquitination inhibitor MG132 (10^−6^ mol⋅mL^−1^). The results showed that MG132 treatment significantly inhibited HNK‐induced Nur77 protein downregulation (Figure 7B). Collectively, these findings suggest that HNK reduces Nur77 protein expression via transcriptional regulation and ubiquitin‐proteasome pathway‐mediated degradation.

## 4 DISCUSSION

Although several recent studies have demonstrated the beneficial effect of honokiol on cardiac hypertrophy, our study evaluates a new molecular mechanism by which honokiol mediates cardio‐protection. The study uncovers a novel molecular mechanism downstream of honokiol action. Pathological myocardial hypertrophy is known to contribute to the pathogenesis of heart failure, a condition that has become increasingly prevalent and lethal worldwide.^40^ Recently, studies have highlighted the critical role of the LKB1/AMPK signalling pathway in the development of pathological myocardial hypertrophy.^41^, ^42^ Our previous research has demonstrated that retinoid X receptor agonists can alleviate cardiomyopathy in streptozotocin‐induced type‐1 diabetes by exerting LKB1‐dependent anti‐fibrotic effects.^43^ This, once again, reinforces the essential role of the LKB1‐AMPK axis in the pathological remodelling of the heart.

In order to investigate the mechanism by which HNK ameliorates pathological myocardial remodelling and cardiac dysfunction, we assessed the impact of HNK intervention on the LKB1‐AMPK pathway both *in vitro* and *in vivo*. Our findings revealed that in Ang II‐induced hypertrophic hearts and NRVMs, the levels of p‐LKB1 and p‐AMPK were diminished, whereas those of p‐p70S6K and ANP were significantly elevated. However, HNK significantly attenuated the effects of Ang II on p‐LKB1, p‐AMPK and p‐p70S6K, resulting in reduced collagen deposition in hypertrophic hearts. These results demonstrate that the modulation of the LKB1‐AMPK‐p70S6K pathway plays a pivotal role in HNK‐mediated mitigation of Ang II‐induced pathological myocardial remodelling.

Nur77, among the three NR4A receptors, is highly expressed in the adult mouse heart.^44^ Numerous stressors, including isoproterenol^44^ and transverse aortic constriction,^45^ have been shown to rapidly increase Nur77 protein expression in the myocardium. Consistent with these findings, our *in vivo* and ex vivo experiments demonstrated that Ang II intervention significantly upregulated the protein levels of Nur77 in the heart. Furthermore, previous study revealed that Nur77 in cardiac fibroblasts promotes the transition of fibroblast‐to‐myofibroblast induced by isoproterenol and transforming growth factor (TGF)‐β, while in myocytes, Nur77 inhibits the ability of myocytes to induce paracrine TGF‐β‐mediated transition of fibroblast‐to‐myofibroblast, thereby maintaining the balance of cardiac fibrotic response ^[39]^. These results highlight the important role of Nur77 in modulating heart function and remodelling in various cardiac pathologies.

The Nur77–LKB1 interaction has been shown to play a role in various physiological and pathological processes. Previous studies have demonstrated that Nur77 specifically binds to LKB1 in the nucleus, which represses the nuclear export of LKB1 and its subsequent phosphorylation.^17^ Conversely, repression of Nur77 binding with LKB1 may have a positive effect on hepatic steatosis.^46^ Recent studies have identified TMPA as a Nur77 antagonist that inhibits the binding of Nur77 to LKB1 and promotes the nuclear export of LKB1 to the cytoplasm and its subsequent phosphorylation, which stimulates downstream molecules associated with lipid metabolism and prevents fatty acid aggregation.^39^ In this study, we demonstrated that Nur77 interacts with LKB1 in the nucleus and that overexpression of Nur77 enhances its binding to LKB1. Our findings indicate that HNK blocks the formation of the Nur77–LKB1 complex, promotes the nuclear export of LKB1 to the cytoplasm and enhances its phosphorylation. This, in turn, promotes the phosphorylation of AMPKα and suppresses the downstream p70S6K. Our data suggest that promoting the dissociation of the Nur77–LKB1 complex in the nucleus may be a vital mechanism by which HNK ameliorates Ang II‐induced myocardial hypertrophy.

As previously mentioned, the interaction between Nur77 and LKB1 in the nucleus of cardiomyocytes, as well as downstream AMPK and p70S6K activities, are directly influenced by the level of Nur77 protein. Additionally, we observed that HNK attenuated the Ang II‐induced increase in Nur77 protein levels. Intracellular protein levels are governed by mRNA transcription and ubiquitination‐proteasome degradation, and we aimed to investigate the mechanisms by which HNK regulates intracellular Nur77 protein levels in this study. We separately examined these two aspects to gain insights into the possible mechanisms. Our findings suggest that HNK inhibits Ang II‐induced Nur77 mRNA transcription and promotes Nur77 protein degradation. However, when the NRVMs were pre‐treated with the protease inhibitor MG132, the pro‐Nur77 protein degradation effect of HNK was significantly reversed. Thus, inhibition of mRNA transcription and promotion of ubiquitinated degradation appear to be critical for HNK to regulate Nur77 protein levels and subsequently affect the LKB1‐AMPK‐p70S6K pathway. In summary, our results suggest that HNK promotes the dissociation of LKB1 from Nur77 through both genomic and non‐genomic effects.

## 5 CONCLUSION

In summary, our study represents the first to demonstrate, through *in vitro* and *in vivo* experimentation, the ability of HNK to alleviate cardiac pathological hypertrophy, fibrosis and heart dysfunction induced by Ang II. The underlying mechanism involves HNK disrupting the interaction between Nur77 and LKB1, resulting in the activation of the LKB1/AMPK signalling pathway. These findings offer novel insights into the modulation of the LKB1‐AMPK pathway by HNK and highlight the potential of HNK as a promising therapy for preventing myocardial hypertrophy.

## AUTHOR CONTRIBUTIONS

**Xiaoyan Lin:** Conceptualization (lead); data curation (lead); formal analysis (lead); funding acquisition (supporting); investigation (lead); methodology (lead). **Hailin Zhang:** Formal analysis (supporting); methodology (supporting). **Yong Chu:** Conceptualization (lead); data curation (lead); formal analysis (lead); funding acquisition (supporting); investigation (lead); methodology (lead); writing – original draft (lead). **Yuze Zhang:** Formal analysis (supporting); methodology (supporting). **Changsheng Xu:** Conceptualization (supporting); supervision (supporting). **Hong Xie:** Conceptualization (supporting); supervision (supporting). **Qinyun Ruan:** Conceptualization (supporting); supervision (supporting). **Jinxiu Lin:** Conceptualization (supporting); supervision (supporting). **Chunkai Huang:** Conceptualization (supporting); supervision (supporting). **Dajun Chai:** Data curation (lead); funding acquisition (lead); project administration (lead); resources (lead); supervision (lead); validation (lead); writing – review and editing (lead).

## FUNDING INFORMATION

This work was supported by grants from the Startup Fund for scientific research, Fujian Medical University (2020QH2032) and was partly supported by the Joint Funds for the Innovation of Science and Technology of Fujian Province (2018Y9088), Natural Science Foundation of Fujian Province of China(2020 J01962) and the Fujian Provincial Health Technology Project (2020QNA052).

## CONFLICT OF INTEREST STATEMENT

The authors declare that they have no competing interests.

## Supporting information


Figure S1–S8.

## Data Availability

The data sets generated and analysed for this study are available from the corresponding author upon reasonable request.
