# Supplementary material for: Honokiol ameliorates angiotensin II‐induced cardiac hypertrophy by promoting dissociation of the Nur77–LKB1 complex and activating the AMPK pathway
Source: J Cell Mol Med. 2023 Nov 20;28(1):e18028. doi: 10.1111/jcmm.18028 (PMC10805491; doi:10.1111/jcmm.18028)
Supplement: Supplementary file 1 — Figure S1–S8. [file JCMM-28-e18028-s001.docx]

**Supplementary materials**

**Figure 1**

**
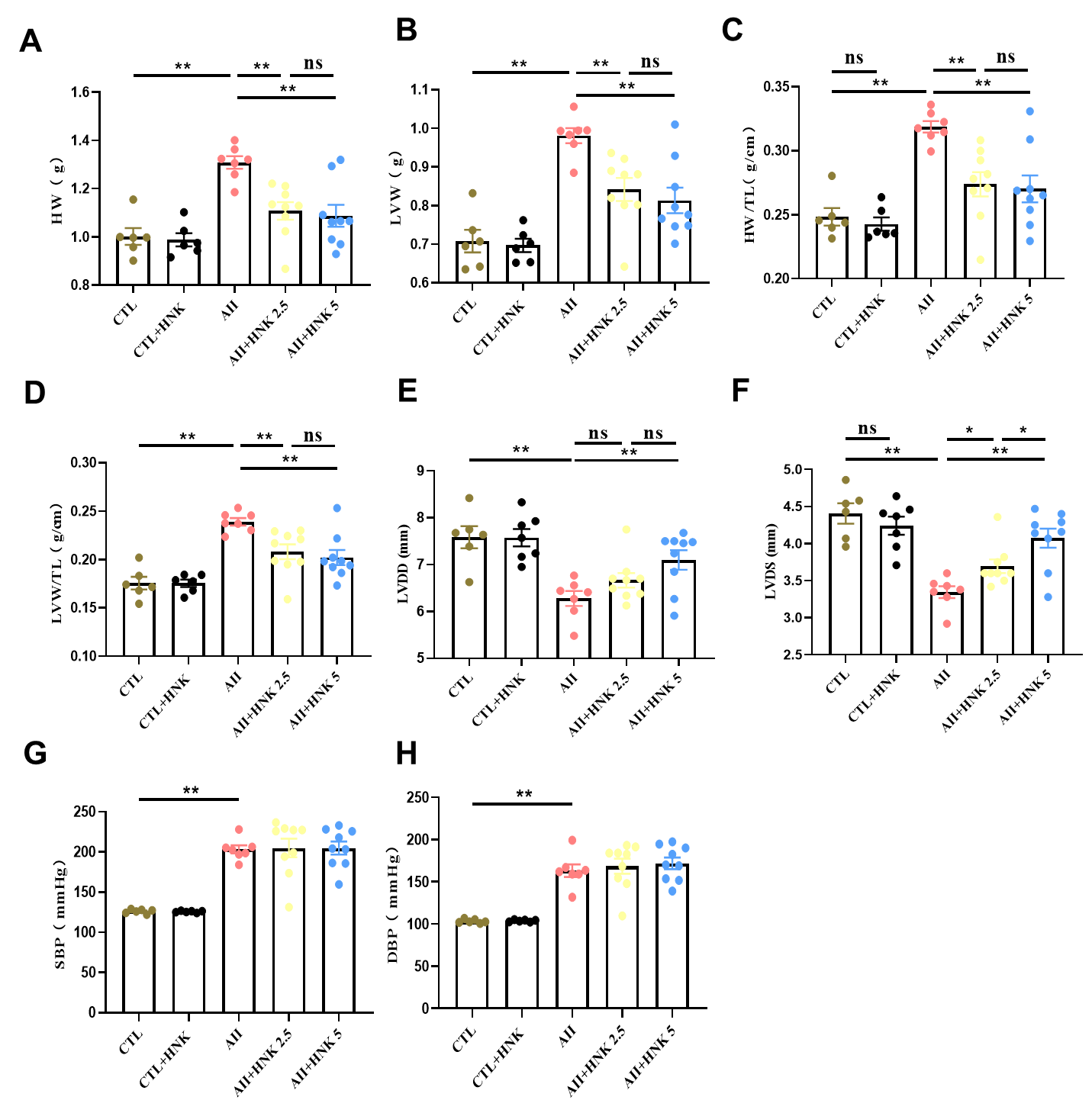
**

**Figure 1 HNK inhibits the cardiac hypertrophic response to Ang II stimulation**

**A–B** Quantitative analysis of heart weight (HW) and left ventricular (LV) weight (LVW) (n = 6–9 rats per group). **C–D** Ratios of HW and LVW to tibia length (n = 6–9 rats per group). **E–F** Echocardiography measurements of LV internal dimensions during diastole (LVDD), LV internal dimensions during systole (LVDS) (n = 6–9 rats per group), at four weeks after implantation of capsules containing Ang II into the subcutaneous area in the backs of the mice. **G–H** Diastolic blood pressure (DBP) and systolic blood pressure (SBP) were measured once a week after implantation of capsules containing Ang II. ns. indicates no statistical significance. ^*^*P* < 0.05 and ^**^*P* < 0.01, as assessed by means of one-way analysis of variance followed by Tukey’s multiple comparison test. Data have been shown as mean ± S.E.M.

**Figure 2**


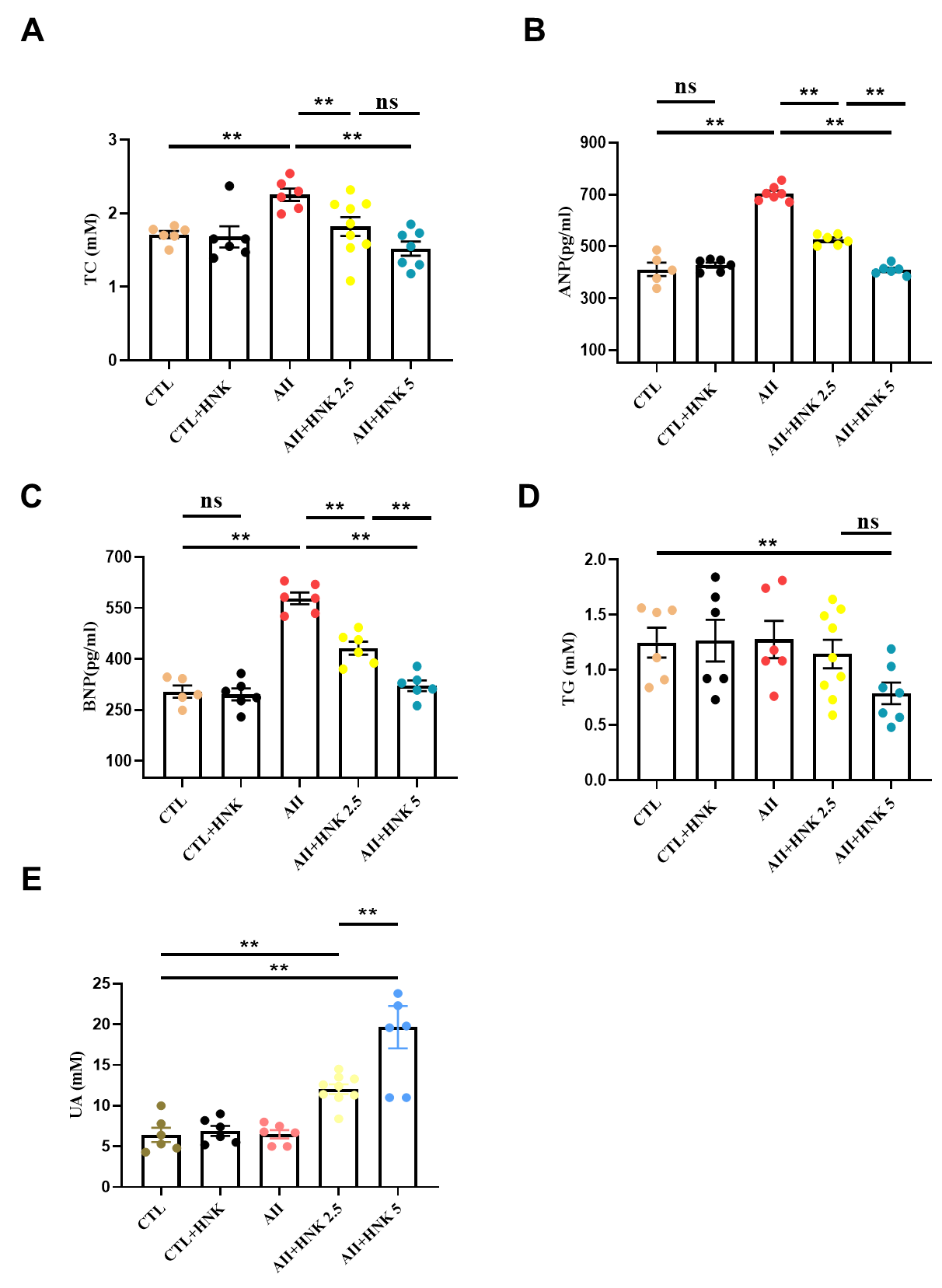


**Figure 2 HNK improves lipid metabolism but increases uric acid levels**

**A–E** Plasma total triglyceride (TG), atrial natriuretic peptide (ANP), brain natriuretic peptide (BNP), cholesterol (TC) and uric acid (UA) concentrations were examined 28 days after Ang II infusion. ns, no statistical significance. ^**^*P* < 0.01, as assessed using one-way ANOVA followed by Tukey’s multiple comparison test. Data have been presented as mean ± S.E.M.


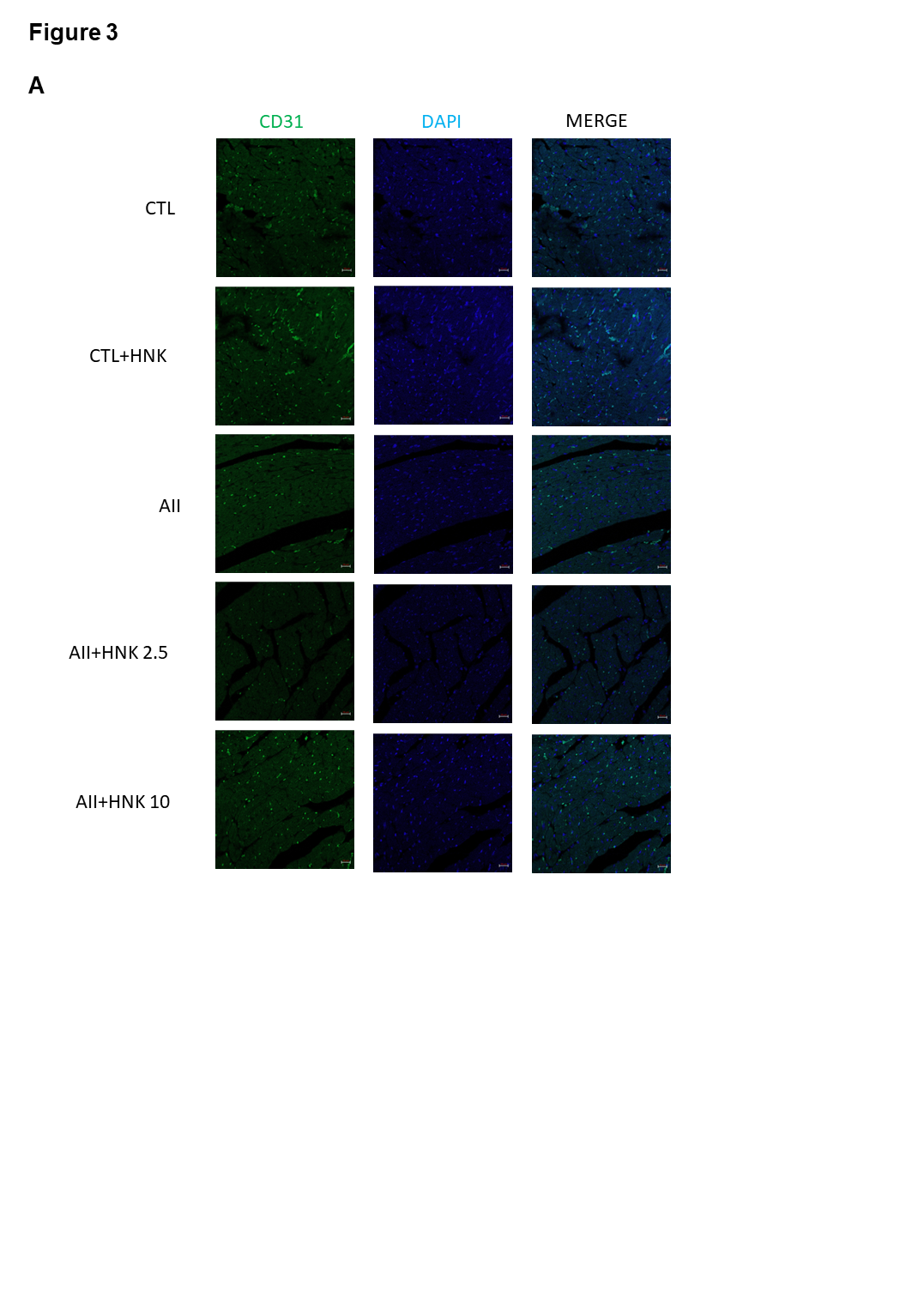


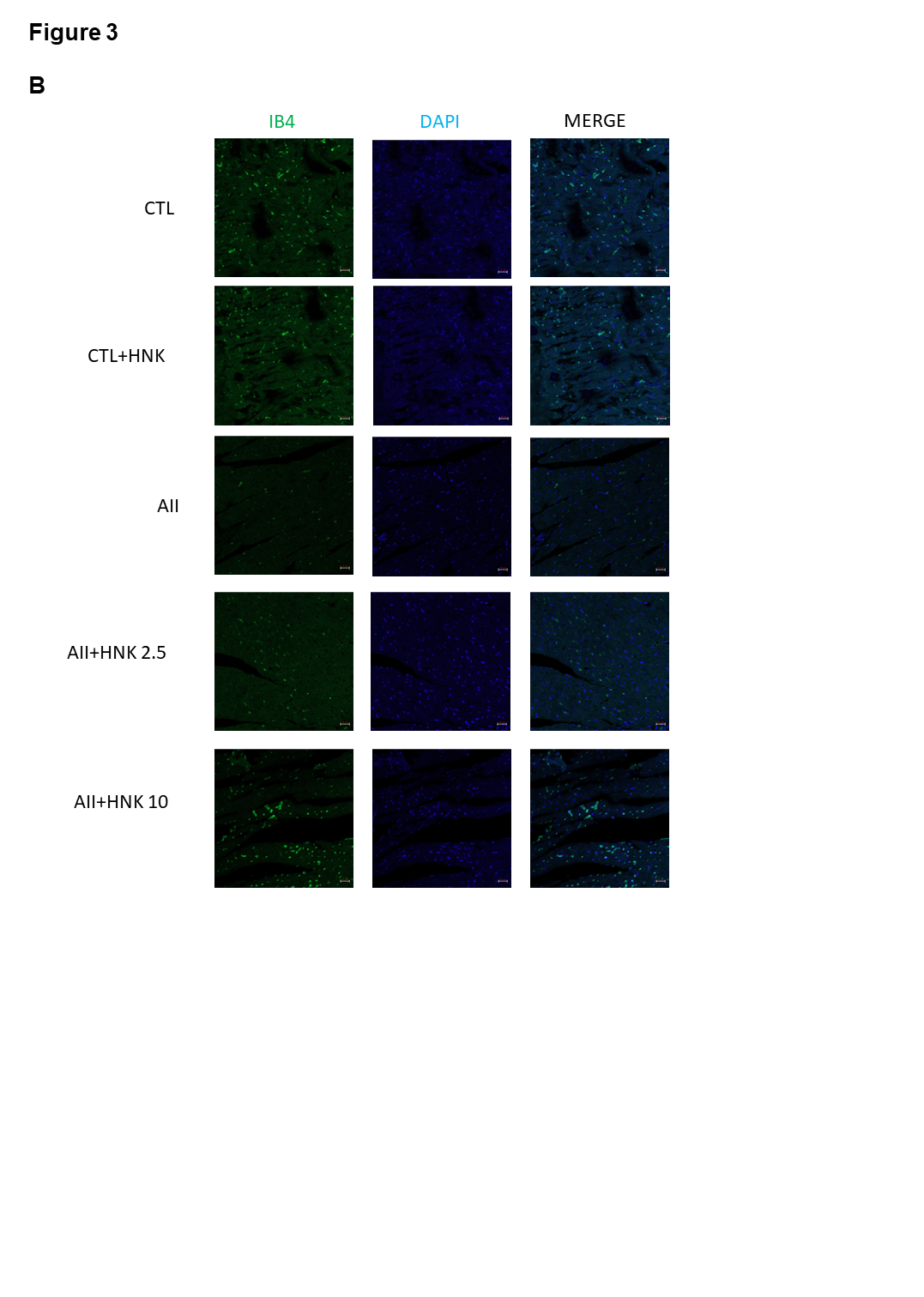


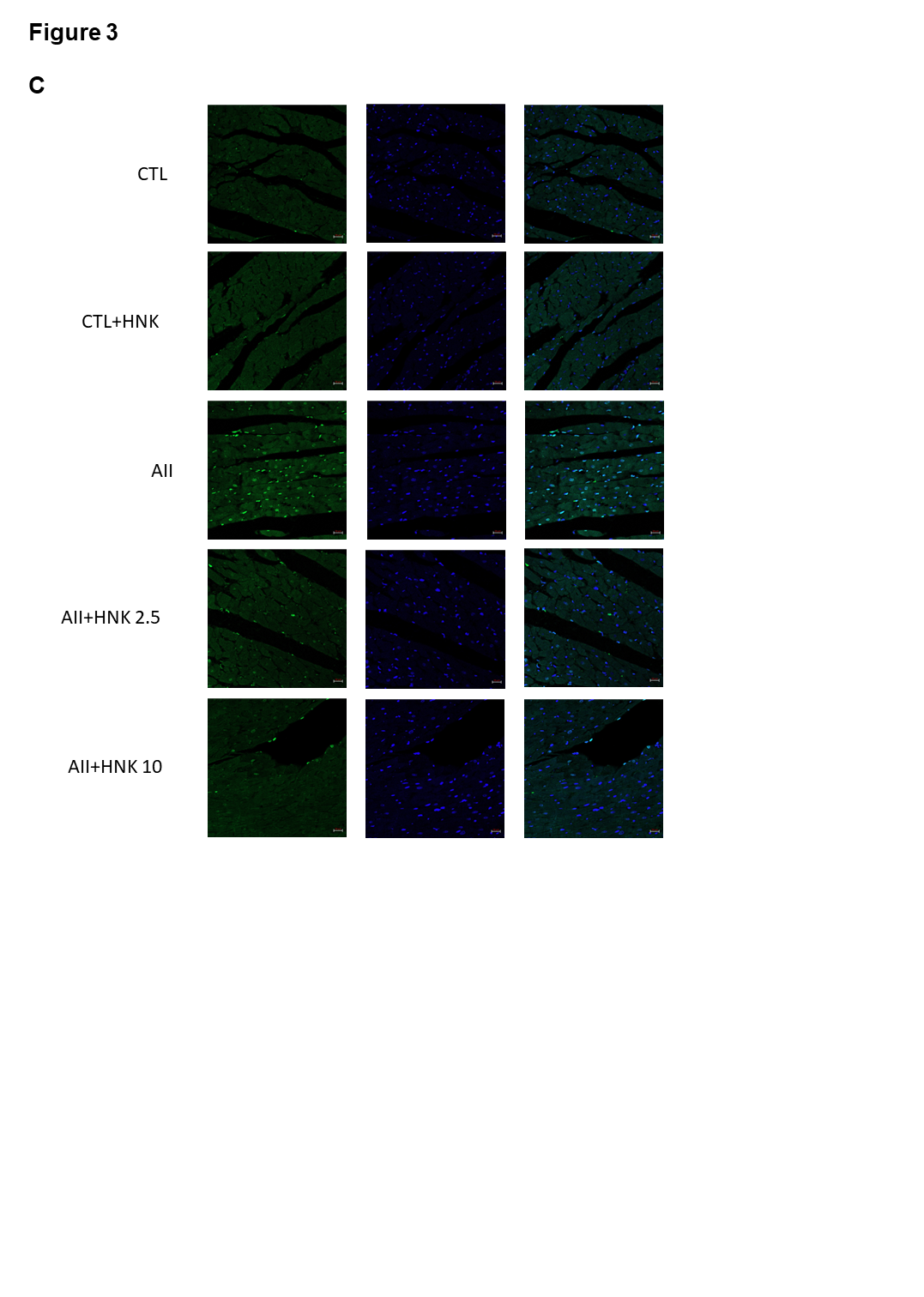


**Figure 3 HNK inhibits cardiomyocyte hypertrophy *in* *vivo* and *in* *vitro***

**A and B** Representative confocal IF images of CD31 and IB4 obtained from capillaries and arteries in LV from CTL, CTL+HNK, AⅡ，AⅡ+HNK2.5 and AⅡ+HNK10 are shown. **B** Representative LV sections stained with TUNEL.DAPI staining denotes nuclei.


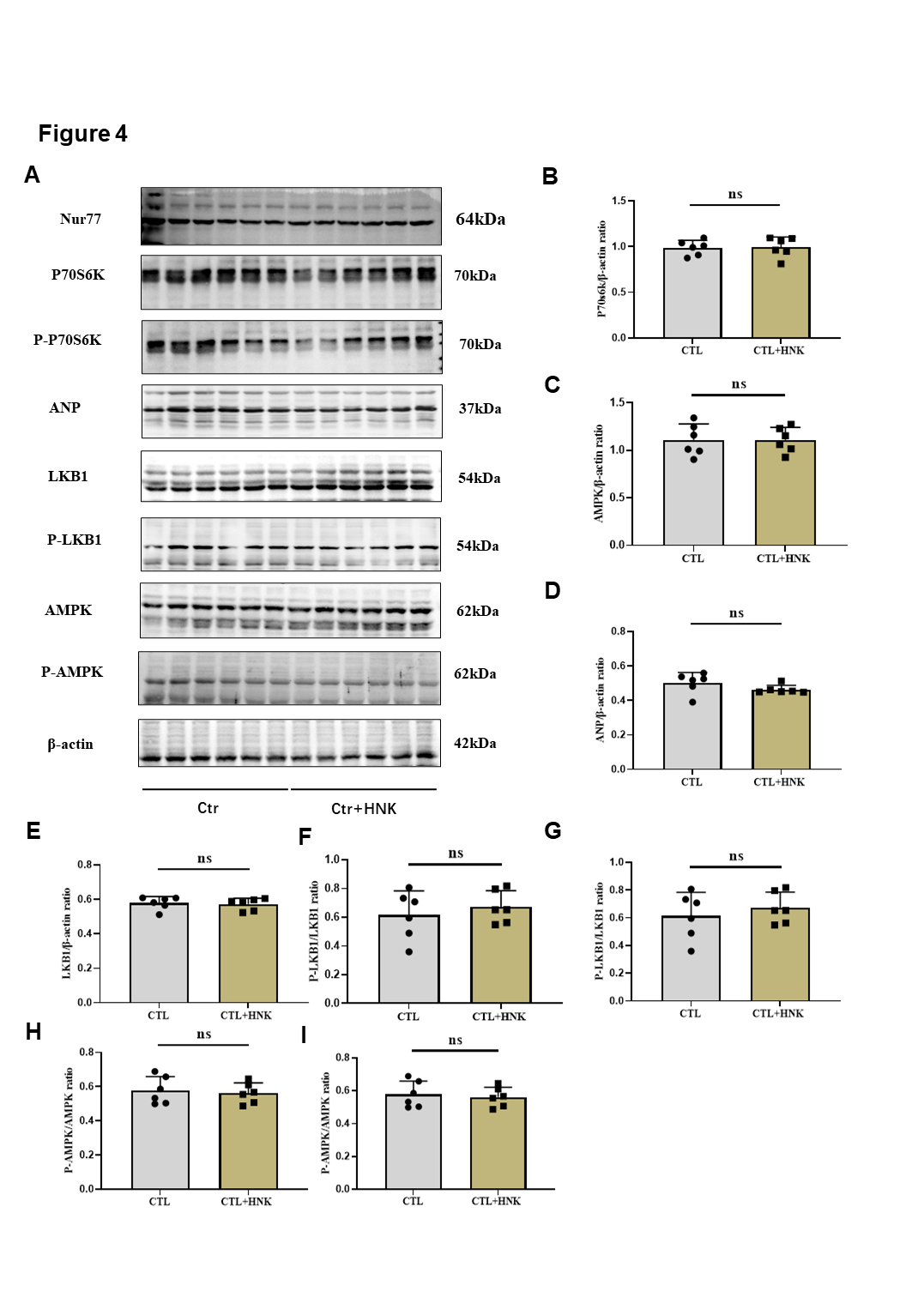


**Figure 4 Examination of the LKB1/AMPK/p70S6k signalling pathway in SD rats treated with HNK alone**

**A** All proteins of the Nur77/p70S6k signalling pathway were examined by means of immunoblotting in myocardial tissue samples from CTL- and HNK-treated rats; quantification analysis of the results is presented in **B–I** (n = 6 rats per group). ns. indicates no statistical significance as assessed using one-way ANOVA followed by Tukey’s multiple comparison test.


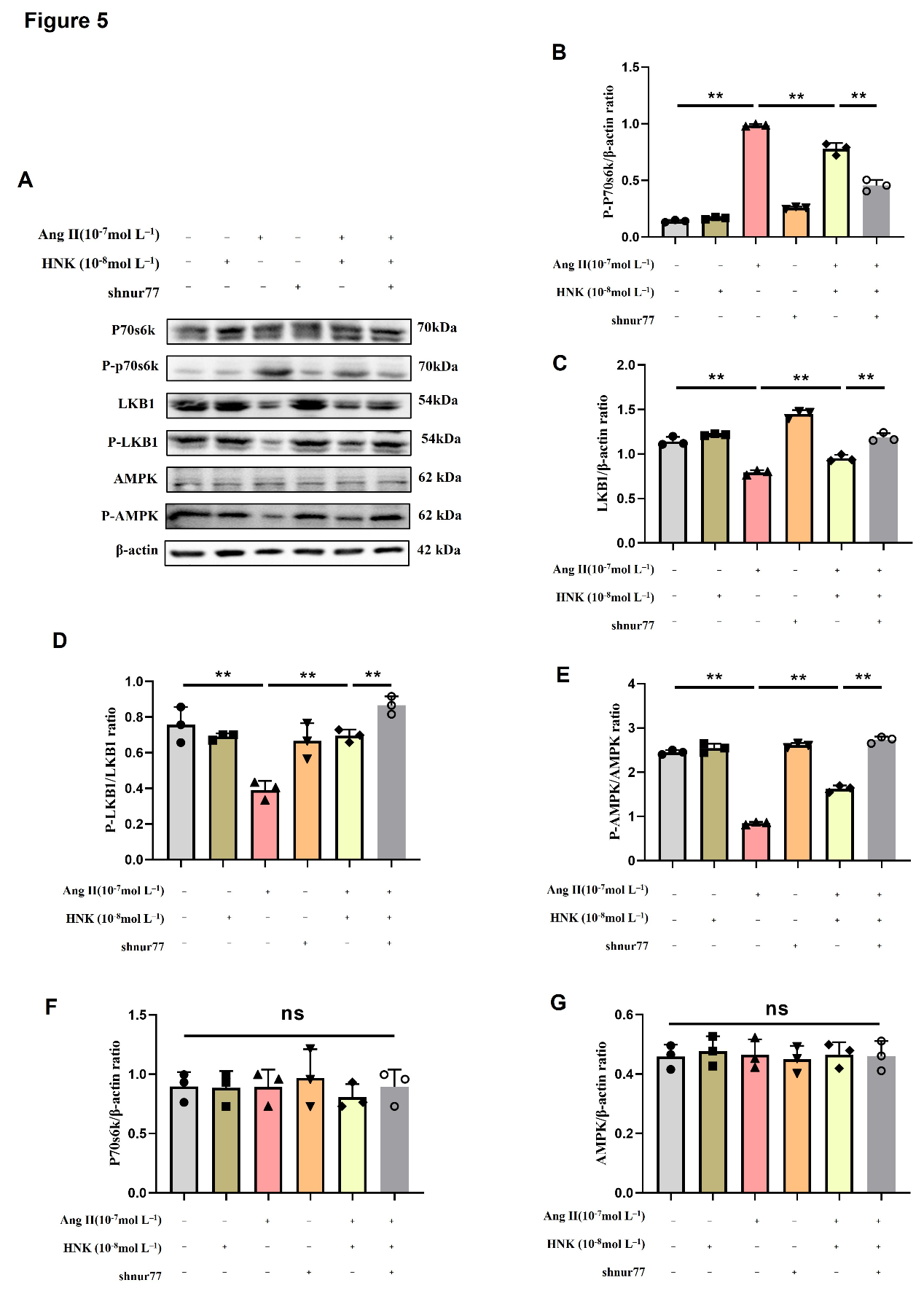


**Figure 5 HNK antagonizes the regulatory effects of Ang II on the LKB1/AMPK/p70S6K pathway in NRVMs cultured *in vitro*.**

**A-G** Representative immunoblots and quantification of the major proteins of the LKB1/AMPK/p70S6K signalling pathway in NRVMs subjected to knock down nur77 and treatment with HNK (10^–8^ mol⋅mL^–1^), followed by activation with Ang II (10^–7^ mol⋅mL^–1^) for 1 h, to induce hypertrophy (n = 3). ns. indicates no statistical significance. ^*^*P* < 0.05 and ^**^*P* < 0.01, as assessed using one-way analysis of variance followed by Tukey’s multiple comparison test. Data have been shown as mean ± S.E.M.


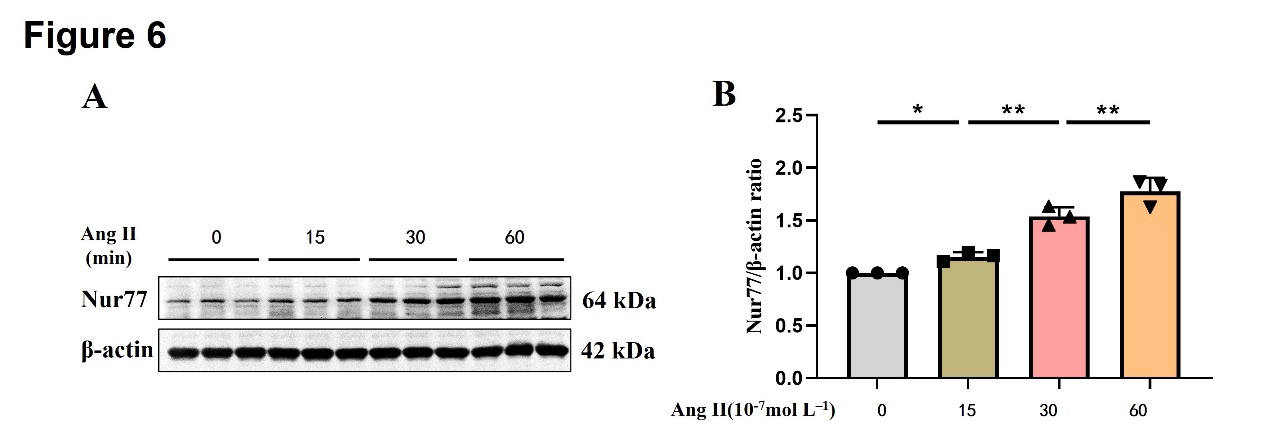


**Figure 6 Expression of Nur77 in NRVMs**

**A–B** Representative immunoblots and quantification analysis of Nur77 protein level in NRVMs treated with Ang II (10^–6^ mol⋅L^–1^), for 0, 15, 30, and 60 min (n = 3). ^*^*P* < 0.05 and ^**^*P* < 0.01, as assessed using one-way ANOVA followed by Tukey’s multiple comparison test.

**
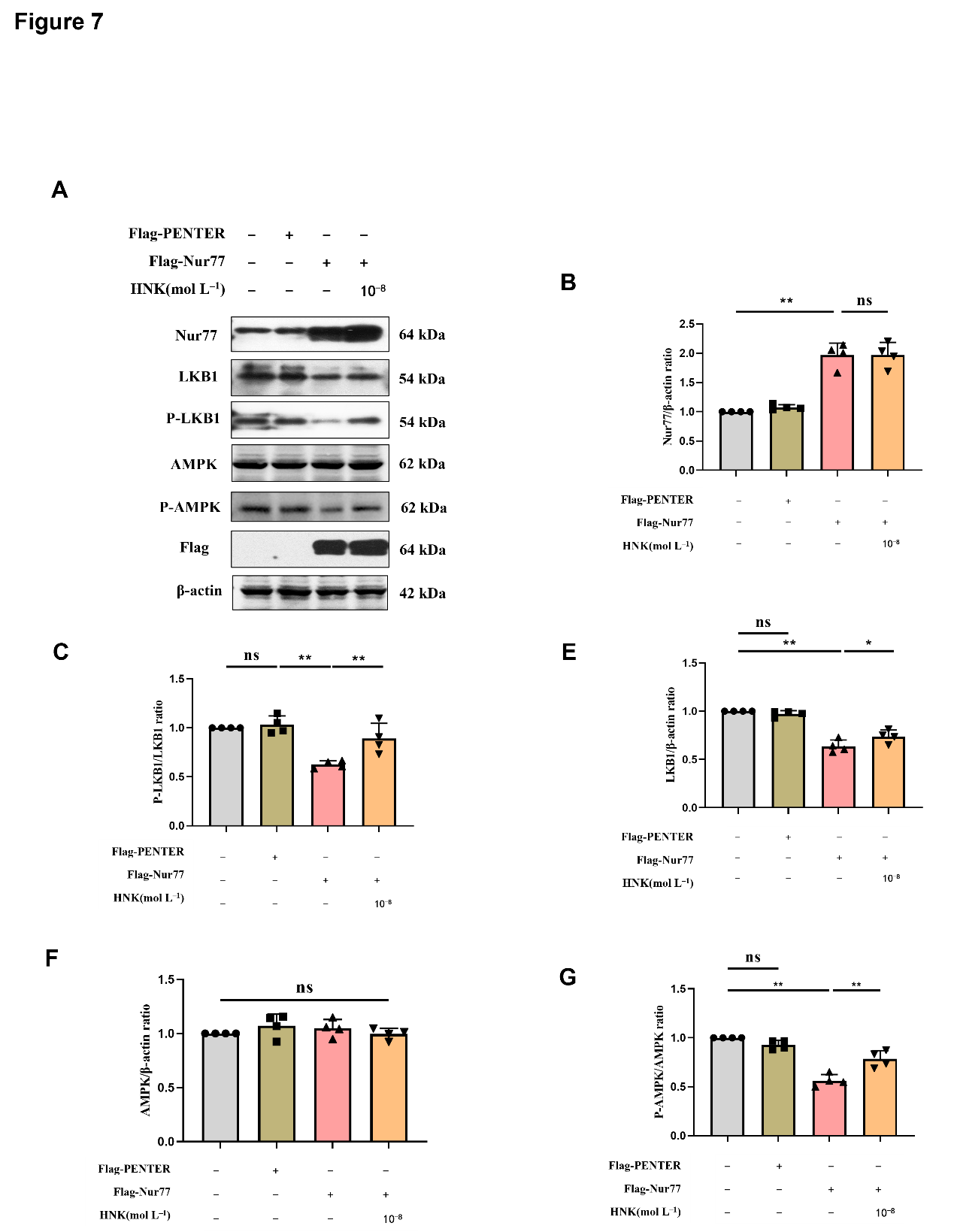
**

**Figure 7 HNK mediates modulation of the LKB1/AMPK signalling pathway by Nur77**

**A–G** 293T cells were treated with HNK for 30 min, following which they were transfected with Flag-His-tagged Nur77 plasmid using Lipofectamine™ 3000. HNK, honokiol; Ang II, angiotensin II. ns. no statistical significance. ^**^*P* < 0.01, as assessed using one-way ANOVA followed by Tukey’s multiple comparison test. Data have been shown as mean ± S.E.M.


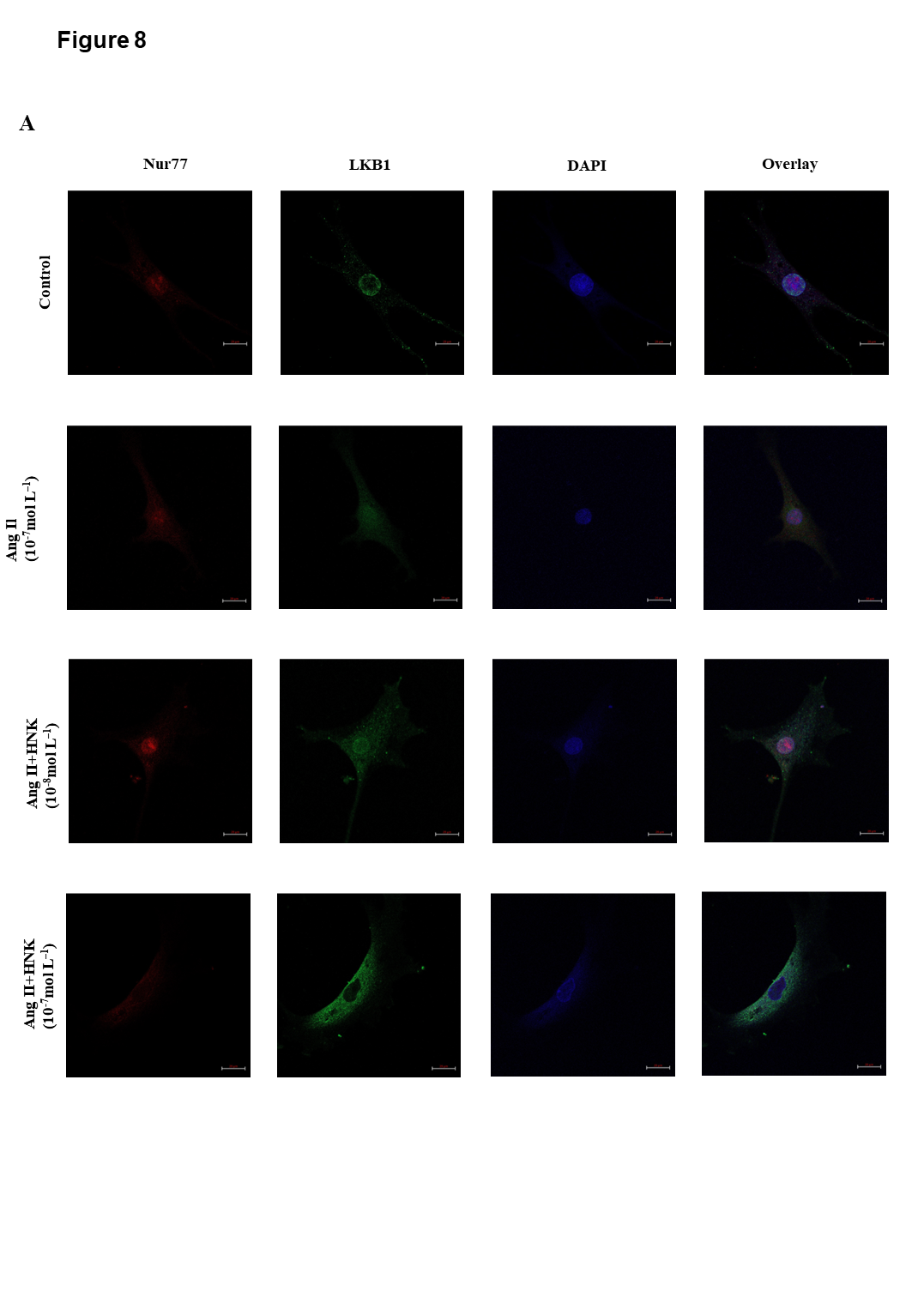


**Figure 8 HNK promotes dissociation of the Nur77-LKB1 complex to activate downstream signalling pathways.**

**A** Subcellular co-localisation of Nur77 and LKB1. Immunofluorescence in the treated NRVMs was examined using confocal fluorescence microscopy. Scale bar: 20 µm (n = 3) **.**
